# Action mechanism of hypoglycemic principle 9-(R)-HODE isolated from cortex lycii based on a metabolomics approach

**DOI:** 10.3389/fphar.2022.1011608

**Published:** 2022-10-21

**Authors:** Yueqiu Liu, Xinyi Hu, Wen Zheng, Lu Zhang, Luolan Gui, Ge Liang, Yong Zhang, Liqiang Hu, Xin Li, Yi Zhong, Tao Su, Xin Liu, Jingqiu Cheng, Meng Gong

**Affiliations:** ^1^ Laboratory of Clinical Proteomics and Metabolomics, Institutes for Systems Genetics, Frontiers Science Center for Disease-Related Molecular Network, National Clinical Research Center for Geriatrics, West China Hospital, Sichuan University, Chengdu, China; ^2^ College of Materials and Chemistry and Chemical Engineering, Chengdu University of Technology, Chengdu, China

**Keywords:** metabolomics approach, 9-(R)-HODE, diabetic therapy, hypoglycemic effect, gut microbiota, SCFAs, DESI-MSI

## Abstract

The 9-(R)-HODE is an active compound isolated from cortex lycii that showed significant hypoglycemic effects in our previous *in vitro* study. In this study, 9-(R)-HODE’s *in vivo* hypoglycemic activity and effect on alleviating diabetic complications, together with its molecular mechanism, was investigated using a metabolomics approach. The monitored regulation on dynamic fasting blood glucose, postprandial glucose, body weight, biochemical parameters and histopathological analysis confirmed the hypoglycemic activity and attenuation effect, i.e., renal lesions, of 9-(R)-HODE. Subsequent metabolomic studies indicated that 9-(R)-HODE induced metabolomic alterations primarily by affecting the levels of amino acids, organic acids, alcohols and amines related to amino acid metabolism, glucose metabolism and energy metabolism. By mediating the related metabolism or single molecules related to insulin resistance, e.g., kynurenine, myo-inositol and the branched chain amino acids leucine, isoleucine and valine, 9-(R)-HODE achieved its therapeutic effect. Moreover, the mediation of kynurenine displayed a systematic effect on the liver, kidney, muscle, plasma and faeces. Lipidomic studies revealed that 9-(R)-HODE could reverse the lipid metabolism disorder in diabetic mice mainly by regulating phosphatidylinositols, lysophosphatidylcholines, lysophosphatidylcholines, phosphatidylserine, phosphatidylglycerols, lysophosphatidylglycerols and triglycerides in both tissues and plasma. Treatment with 9-(R)-HODE significantly modified the structure and composition of the gut microbiota. The SCFA-producing bacteria, including Rikenellaceae and Lactobacillaceae at the family level and *Ruminiclostridium 6*, Ruminococcaceae *UCG 014*, *Mucispirillum*, *Lactobacillus, Alistipes* and *Roseburia* at the genus level, were increased by 9-(R)-HODE treatment. These results were consistent with the increased SCFA levels in both the colon content and plasma of diabetic mice treated with 9-(R)-HODE. The tissue DESI‒MSI analysis strongly confirmed the validity of the metabolomics approach in illustrating the hypoglycemic and diabetic complications-alleviation effect of 9-(R)-HODE. The significant upregulation of liver glycogen in diabetic mice by 9-(R)-HODE treatment validated the interpretation of the metabolic pathways related to glycogen synthesis in the integrated pathway network. Altogether, 9-(R)-HODE has the potential to be further developed as a promising candidate for the treatment of diabetes.

## 1 Introduction

Approximately 537 million adults worldwide live with diabetes, with a prevalence of 10%, which will reach 643 million by 2030. Moreover, the reported number of patients with diabetes does not include those who are undiagnosed, amounting for 44.7% of the uncounted individuals (International Diabetes Federation, 2021). Diabetes caused 6.7 million deaths in 2021, with one death occurring every five seconds. Diabetes introduces a huge burden to the public health system due to its high morbidity, multiple complications, and high expenditure. A 316% increase in costs on diabetes occurred over the last 15 years (International Diabetes Federation, 2021). The currently available drugs for the treatment of diabetes include insulin and different types of oral hypoglycemic agents, which can have one or more side effects (Carpio and Fonseca, 2014). Thus, discovering new drugs with excellent efficiency and minimal side effects could ease the huge health burden caused by diabetes.

Due to their advantages of no systemic side effects, α-glucosidase inhibitory drugs have been used as the first-line oral hypoglycemic drugs since the 1990s. The (9*R*,10*E*,12*Z*)-9-hydroxy-10,12-octadecadienoic acid (9-(R)-HODE, Figure 10) is an active compound that was isolated from cortex lycii in our previous study and showed considerable α-glucosidase inhibitory activity in an *in vitro* study as a noncompetitive inhibitor (Liu et al., 2021). However, whether 9-(R)-HODE is responsible for the *in vivo* therapeutic effect and underlying mechanism of 9-(R)-HODE on diabetes has not been elucidated.

In this study, dynamic fasting blood glucose, postprandial glucose, body weight, biochemical parameters, and tissue pathological changes were monitored to confirm the hypoglycemic activity and attenuation effect of 9-(R)-HODE. Metabonomics, as a powerful approach, can provide a comprehensive assessment of the global metabolic regulation of organisms corresponding to internal or external influences. In recent years, the development of analytical platform liquid chromatography-tandem mass spectrometry (LC‒MS/MS), combined with biostatistics, has allowed for unbiased determination of various metabolites on a massive data scale. Today, integrated metabolomics has been widely applied in drug discovery, including natural active principle screening and mechanism of action exploration in disease treatment (Liu et al., 2021; Chen et al., 2022). LC‒MS/MS-based metabolomic and lipidomic analyses were performed to explore the underlying mechanism of action of 9-(R)-HODE at the molecular level. The recent development of desorption electrospray ionization-mass spectrometry imaging (DESI‒MSI), an advanced ambient ionization technique, allows for the spatial mapping of biomolecules in tissue sections from sample surfaces (Thoma, 2017). DESI‒MSI was applied in the present study to verify the above metabolomics results by further exploring the presence and spatial distribution patterns of differential molecules among all the mouse groups. Short-chain fatty acids (SCFAs) are closely related to lipids (Jäger et al., 2007; Tang et al., 2020), glucose, energy metabolism (Fujikawa et al., 2010; Barrera et al., 2011) and inflammatory processes (Ohira et al., 2013; Halnes et al., 2017), and they themselves are affected by SCFA-producing bacteria. *In vivo* experiments revealed that hypoglycemic drugs or substances might exert hypoglycemic effects by altering the abundance of SCFA-producing bacteria (Zhang and Hu, 2020; Xia et al., 2021). In this study, the components of the gut microbiota in the mouse colon contents were analyzed by 16S rRNA gene sequencing, and the SCFA abundances in the colon contents were determined by gas chromatography‒mass spectrometry (GC‒MS) to explore the possible multiple action mechanisms of 9-(R)-HODE.

Altogether, the *in vivo* therapeutic effect and underlying mechanism of 9-(R)-HODE on diabetes were elucidated in this study.

## 2 Materials and methods

### 2.1 Chemicals and reagents

The compound 9-(R)-HODE (purity ≧99%) was purchased from HitGen Inc. (Chengdu, China). Additional information on the reagents used is provided in the Chemicals and reagents section in the Supporting Information.

### 2.2 Animal experiments

All animal experiments were performed in compliance with the guidelines of the Animal Care and Use Committee of Sichuan University (Committee approval #2021875A). Mature male C57/BL6 mice weighing between 22 and 25 g were used in this study. The mice had free access to water and food and were housed in standard cages at room temperature (22–24°C) with a 12-h light-dark cycle and approximately 40–60% humidity. The mice were adaptively fed for 7 days, and thereafter, 40 mice were fasted for 8 h and continuously injected with streptozotocin (STZ, 50 mg/kg in sodium citrate-citric acid buffer) for 5 days, while five normal mice were randomly picked as the control group by injecting them with the same amount of sodium citrate-citric acid buffer (0.1 M, pH 4.5). Mice with fasting blood glucose (FBG) ≥13.9 mol/L as maintained for 7 days were considered diabetic mice. The mice that met the above criteria were randomly assigned to three groups (five to six mice per group), namely, the model, 9-(R)-HODE treatment and positive groups. All the mouse groups were subsequently fed a normal diet. The 9-(R)-HODE was dissolved and uniformly dispersed in 0.5% carboxymethyl cellulose sodium (CMC-Na) and administered at a concentration of 25 mg/kg/day, while mice in the positive group were treated with acarbose at 10 mg/kg/day (Mohamed et al., 2015). The dosage of 9-(R)-HODE was determined based on our pre-experiments. The model and control group mice received equal amounts of 0.5% CMC-Na solvent. All treatments were administered for 25 days. Body and food weights were recorded daily. FBG and 0.5-, 1.5- and 2.0-h postprandial glucose (PPG) were determined every 5 days by feeding the mice corn starch (1 g/kg). Ultimately, the mice were fasted for 12 h, and blood was collected *via* the eye in tubes containing ethylenediaminetetraacetic acid (EDTA) under general anesthesia with sodium pentobarbital (30 mg/kg) before sacrifice by cervical dislocation. The tissues were removed for subsequent metabolomics and lipidomics study. The fresh colon content was obtained and preserved in liquid nitrogen for subsequent metabolite profiling and microbiota analysis.

### 2.3 Biochemical assays

Mouse plasma was obtained by centrifuging the blood at 2000 rpm (8°C) for 10 min. The plasma biochemical parameters, including total cholesterol (TC), triglyceride (TAG), low-density lipoprotein cholesterol (LDL-C), high-density lipoprotein cholesterol (HDL-C), total protein (TP), alanine transaminase (ALT), aspartate transaminase (AST), uric acid (UA), urea (UREA) and creatinine (CRE), were tested with an automatic analyzer (Secomam, Alès, France), and the results are summarized in Figure 2.

### 2.4 Histological study

The kidneys and livers of the mice from all groups were obtained and soaked in 10% buffered formalin for 48 h. Thereafter, the samples were dehydrated, fixed in paraffin and sliced into 4-µm sections for hematoxylin-eosin (H&E) staining. The sections were observed with a GS3-U3-51S5M-C microscope (Point Grey, Richmond, Canada).

### 2.5 Plasma sample preparation

The plasma samples were prepared by extraction, centrifugation, drying and redissolution, and the detailed information is described in the Supporting Information.

### 2.6 Tissue sample preparation

For metabolomics, 10 mg of each tissue (liver, kidney and muscle) sample, 200 µl of precooled MeOH/H_2_O (4:1) solution, and six steel balls were mixed and homogenized 4 times at 4°C (30 s each time). Then, 800 µL of spiked methanol solution was added and swirled for 3 min at 1,500 rpm (4°C). The supernatant was collected after centrifugation. Another 500 µl of MeOH/H2O (4:1) solution was added to the residue for the second extraction. The supernatant was collected and combined with the former supernatant for vacuum drying. For lipidomics, 10 mg of the tissue was processed with the same protocol of homogenization. Then, 150 µl of spiked methanol solution was added and swirled for 3 min at 1,500 rpm (4C). MTBE (1,000 µl) was added, vortexed, and placed in the dark for 30 min. Thereafter, 250 µl of double deionized water was added, vortexed, and incubated in the dark for 10 min. After centrifugation, 800 µl of supernatant was transferred into an EP tube and vacuum dried for 3 h. Detailed information is provided in the Supporting Information.

### 2.7 Colon content sample preparation

For metabolomics, 60 mg colon content was added to 400 µl water and mixed. Another 800 µl acetonitrile/methanol (1:1) solution was added, vortexed, sonicated for 60 min at 4°C, and placed at -20°C for 60 min. Thereafter, the mixture was centrifuged (13,000 g, 4°C, 15 min). Finally, the supernatant was collected and vacuum dried.

### 2.8 UPLC‒MS/MS conditions for plasma and faeces metabolite profiling

The aqueous-soluble metabolite analysis of plasma and faeces was performed on an Ultimate 3,000 rapid separation liquid chromatograph coupled with a Q Exactive Plus Q-Orbitrap HRMS. For lipidomics, metabolic profiling analysis was performed on a Shimadzu LC-30A liquid chromatograph coupled with an AB Sciex 5,500 triple quadrupole mass spectrometer. The conditions for the chromatographic separation and the ion source parameters are described in the Supporting Information.

### 2.9 UPLC‒MS/MS conditions for tissue metabolite profiling

Tissue metabolic profiling analysis was performed on a Shimadzu LC-30A liquid chromatograph coupled with an AB Sciex 5,500 triple quadrupole mass spectrometer. For metabolomics, a total of 228 metabolites (99 in positive mode and 128 in negative mode) chosen for the targeted analysis represented the major metabolic pathways in the diabetic alleviation effect when using the multiple reaction monitoring mode. For lipidomics, a total of 1,251 lipids (635 in positive mode and 616 in negative mode) representing major metabolic pathways were detected. The conditions for the chromatographic separation and the ion source parameters are described in the Supporting Information.

### 2.10 Gut microbiota analysis

Bacterial genomic DNA was extracted using a DNA Extraction Kit (Magen Biotech, Guangzhou, Guangdong, China) following the manufacturer’s instructions. For bacterial diversity analysis, V3-V4 variable regions of 16S rRNA genes were amplified with universal primers 343 F (5′- TACGGRAGGCAGCAG -3′) and 798 R (5′- AGG​GTA​TCT​AAT​CCT-3′). Raw FASTQ files were quality filtered using Trimmomatic (v0.32, http://www.usadellab.org/cms/index.php?page=trimmomatic), FLASH (v1.2.7, http://ccb.jhu.edu/software/FLASH) and QIIME software (v1.8.0, http://drive5.com/usearch/). Clean reads were subjected to primer sequence removal and clustering to generate operational taxonomic units (OTUs) using Vsearch software (https://github.com/torognes/vsearch) with a 97% similarity cutoff. All representative reads were annotated and aligned against the Silva database (confidence threshold was 70%) using the RDP classifier (http://rdp.cme.msu.edu/). The relative proportion of each OTU was annotated at the phylum, class, order, family, genus and species levels. Alpha and beta diversity and principal component analysis were applied to reveal the diversity of bacteria among the different samples. The relative abundances of biomarkers among mice of the different groups were analyzed with the linear discriminant analysis (LDA) effect size (LEfSe) method (https://huttenhower.sph.harvard.edu/galaxy/).

### 2.11 Measurement of SCFAs

SCFAs in colon content were extracted with 5 mM NaOH containing 0.5 μL/ml caproic acid-d3 as an internal standard and derivatized by propyl chloroformate. The derivatives were separated with *n*-hexane. Authentic standards of SCFAs (acetic acid, propionic acid and butyric acid) and internal standard caproic acid-d3 were all obtained from Sigma‒Aldrich (St. Louis, MO, United States). A mixture of the above SCFA standards was dissolved in 5 mM NaOH and processed in the same way. The derivatives were analyzed using an Agilent 7890A gas chromatography system coupled to an Agilent 5975C mass spectrometric detector (Agilent Technologies, Santa Clara, CA), and the detailed conditions are described in the Supporting Information.

### 2.12 Glycogen content determination

The glycogen content in the mouse livers of different groups was determined using a glycogen assay kit (Solarbio, Beijing, China) according to the manufacturer’s instructions. Briefly, 100 mg of liver was added to 750 µL extraction solvent, placed in a boiling water bath and boiled for 20 min with shaking once every 5 min. The volume of the mixture was fixed to 5 ml with distilled water and centrifuged at 8,000 rpm for 10 min, and the supernatant was removed for testing. The samples and glycogen assay working solution were added to the reaction solution, and the absorbance value was measured at 620 nm.

### 2.13 Statistical analysis

The raw metabolic profile data were imported into MultiQuant (v2.0.3) for integration. The metabolites of plasma were identified based on the available reference standards in our lab and web-based resources. The pretreated data were further processed in R software (v3.5.1, Vienna, Austria, https://www.r-project.org/), where the heatmaps were drawn. Subsequently, a principal component analysis (PCA) and partial least squares discrimination analysis (PLS-DA) were performed after median normalization to discriminate among samples with different treatments. MetaboAnalyst 5.0 (Quebec, Canada, https://www.metaboanalyst.ca/home.xhtml) was used to perform the metabolic pathway analysis. The Wilcox test was performed to compare the data between two groups, and the Kruskal‒Wallis test was performed to investigate alterations among multiple groups. The detailed information is described in the Supporting Information.

### 2.14 Desorption electrospray ionization-mass spectrometry imaging analysis

Liver, kidney and muscle frozen tissues from all treatment groups were sectioned at a thickness of 8 µm. The mass spectrometry imaging profiles were performed using a DESI (2D, Indianapolis, Prosolia, United States) source coupled to an MS (Synapt G2-Si, Waters, Milford, United States). DESI‒MSI was performed in both negative and positive modes. The relative parameters are described in the Supporting Information.

## 3 Results

### 3.1 Effects of 9-(R)-HODE on FBG, PPG and body weight

**FIGURE 1:**
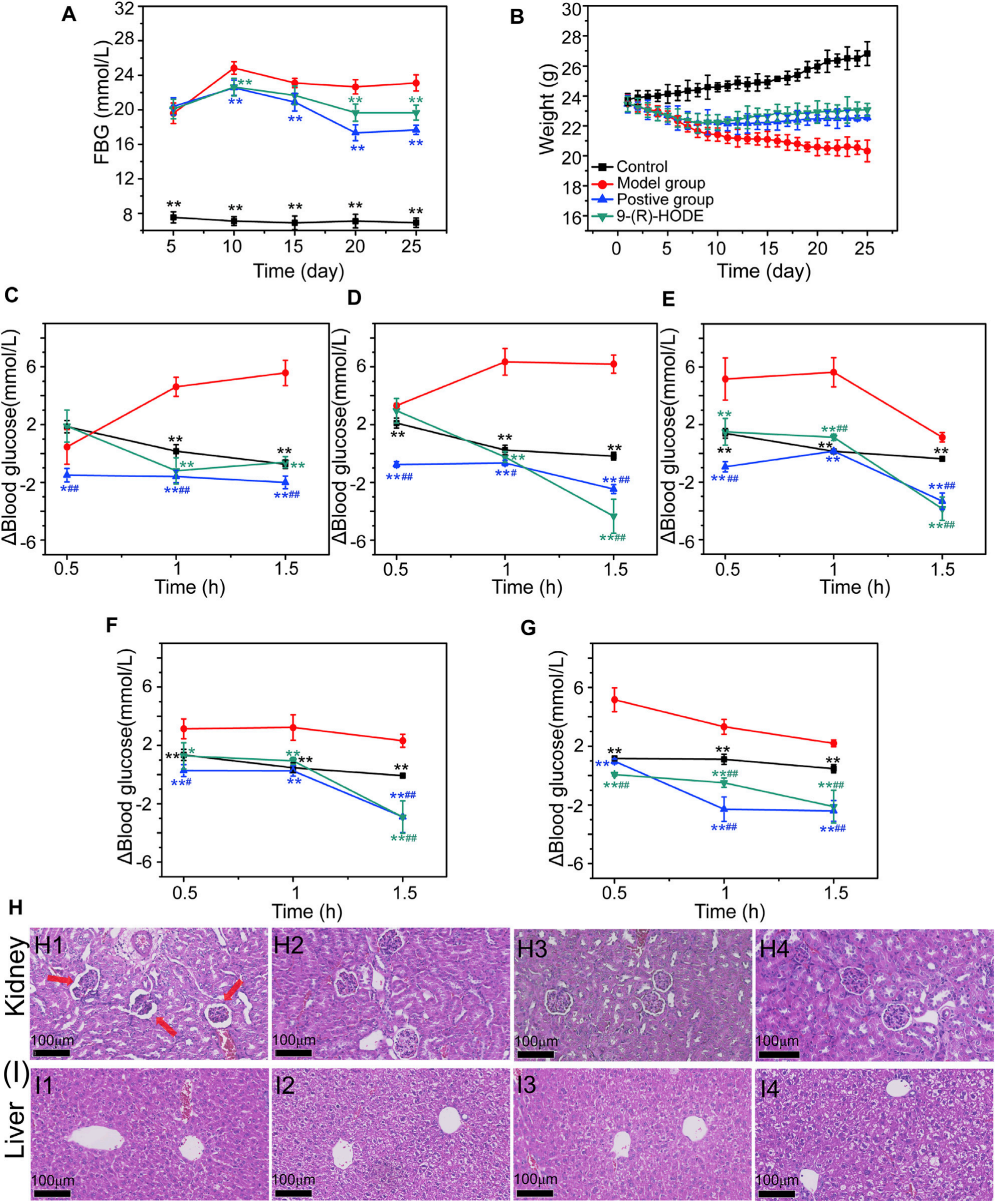
**(A–B)** Effects of 9-(R)-HODE on FBG **(A)** and body weight **(B)**. **(C–G)** Effects of 9-(R)-HODE on PPG tested on Day 5 **(C)**, Day 10 **(D)**, Day 15 **(E)**, Day 20 **(F)**, and Day 25 **(G)**. **(H,I)** Effects of 9-(R)-HODE on kidney **(H)** and hepatic **(I)** pathological changes by using the H&E staining method; comparison between the model group (H1, I1), control group (H2, I2), 9-(R)-HODE treatment group (H3, I3) and positive group (H4, I4); *p < 0.05, **p < 0.01, significantly different from the model group; *#p* < 0.05, *##p* < 0.01, significantly different from the control group.

The dynamic monitoring of FBG is presented in Figure 1A. The diabetic mouse model had a significantly higher FBG value than the normal mouse model, which indicated that the diabetic mouse model was successfully established. Within the first 10 days, compared with the control group, the FBG levels increased dramatically in all the different treatment groups. However, compared with the FBG levels in the model group, those in the group with 9-(R)-HODE treatment, as well as the positive treatment, gradually decreased from Day 15.

Dynamic changes in PPG were monitored by recording the PPG values every half hour for 1.5 h, and the results are presented in Figures 1C–G. The elevated blood glucose level change of PPG (Δblood glucose level: PPG-FBG) in both the positive and 9-(R)-HODE treatment groups was significantly lower (*p* < 0.01) than that in the model group after 1 h of intaking test food, which confirmed their effect on regulating postprandial blood glucose.

Moreover, the effect of 9-(R)-HODE on alleviating weight loss in diabetic mice can be observed in Figure 1B. The body weights of all treatment groups significantly decreased within the first several days, and these changes were reversed from approximately Day 10, while those in the model group continuously decreased (Figure 1B).

### 3.2 Effects of 9-(R)-HODE on biochemical parameters

**FIGURE 2:**
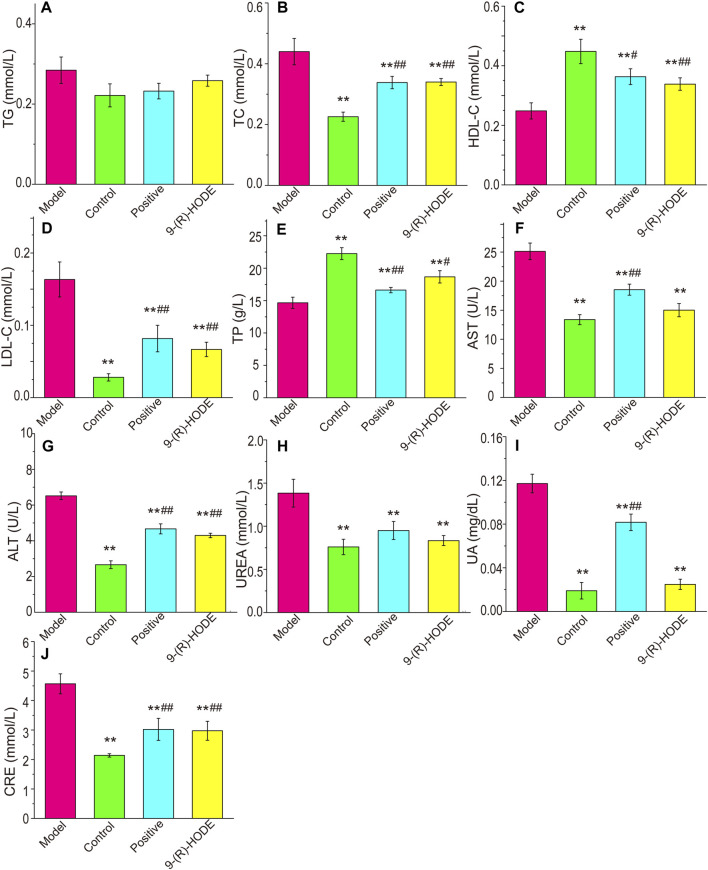
Effects of 9-(R)-HODE on TAG **(A)**, TC **(B)**, HDL-C **(C)**, LDL-C **(D)**, TP **(E)**, AST **(F)**, ALT **(G)**, UREA **(H)**, UA **(I)** and CRE **(J)** plasma levels of mice; *p < 0.05, **p < 0.01, significantly different from the model group; *#p* < 0.05, *##p* < 0.01, significantly different from the control group.

The plasma biochemical parameters, including TAG, TC, LDL-C, HDL-C, TP, AST, ALT, CRE, UA and UREA, are summarized in Figure 2. The TC, LDL-C, AST, ALT, CRE, UA and UREA contents in the model group were significantly higher than those in the control group (*p* < 0.01). The TP and HDL-C contents in the model group were significantly lower than those in the control group (*p* < 0.01). After dosing, the above indices displayed a reverse regulation toward normal levels. It should be noted that although both AST and ALT showed statistical difference among groups, the variations might be due to the fluctuation within the range of normal values, and thus the significance of the above results of these two biochemical parameters is needed to be further verified.

### 3.3 Effects of 9-(R)-HODE on pathological tissue changes

The histopathological analysis of the hepatic and renal tissues from diabetic mice was processed by H&E staining. Of these tissues, pathological changes were observed only in renal tissue compared with the normal control (Figure 1H). Representative HE-stained sections of renal tissues are displayed in Figure 1H. A number of glomeruli tended to atrophy in the model mice (Figure 1H1). After 25 days of treatment, the 9-(R)-HODE treatment group displayed no renal lesions compared with the normal control group, which indicated its identical renal protective efficacy with acarbose (Figures 1H2–1H4).

### 3.4 Metabolomic changes induced by 9-(R)-HODE

**FIGURE 3:**
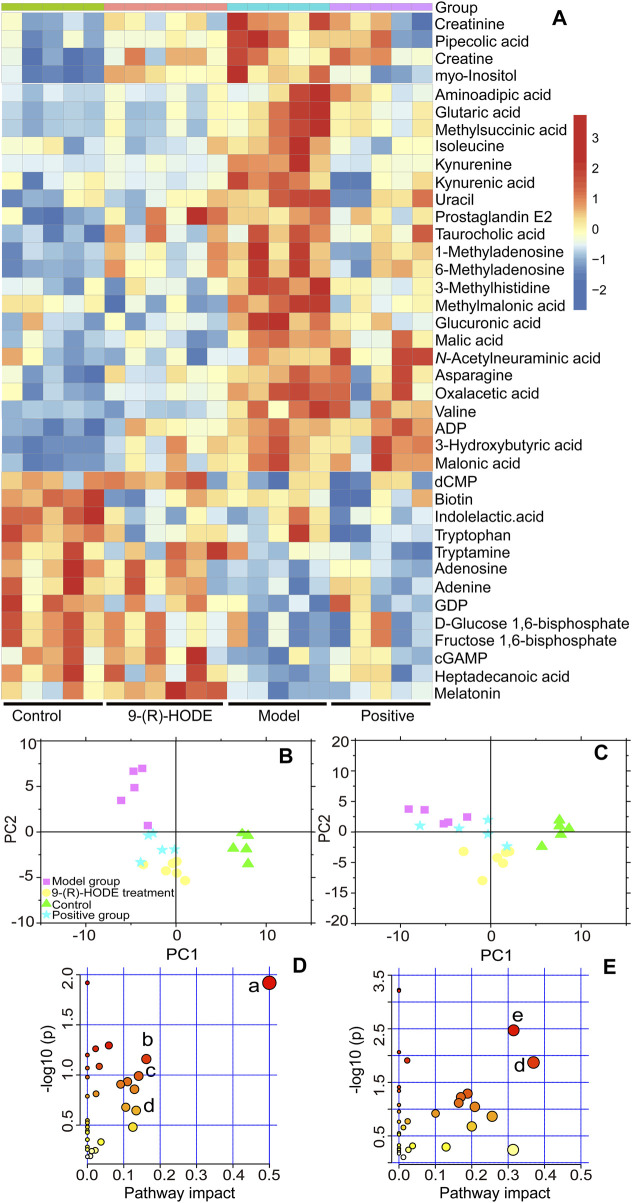
**(A)** Representative extracted heatmap of differential aqueous-soluble metabolites in the liver. The color scale illustrates the relative abundances across the samples. Blue indicates metabolites that were significantly downregulated, while red indicates significantly upregulated metabolites. **(B, C)** PLS-DA score plots of PC1 vs. PC2 based on the LC‒MS/MS data of aqueous-soluble metabolites in liver **(B)** and muscle **(C)**. **(D)** Pathway analysis of metabolites in the liver. The node color is based on its *p* value, and the node radius is determined based on their pathway impact values, **(a)** ascorbate and aldarate metabolism; **(b)** citrate cycle; **(c)** lysine degradation; **(d)** and tryptophan metabolism. **(E)** Pathway analysis of metabolites in muscle. **(e)** Alanine, aspartate and glutamate metabolism.

**FIGURE 4:**
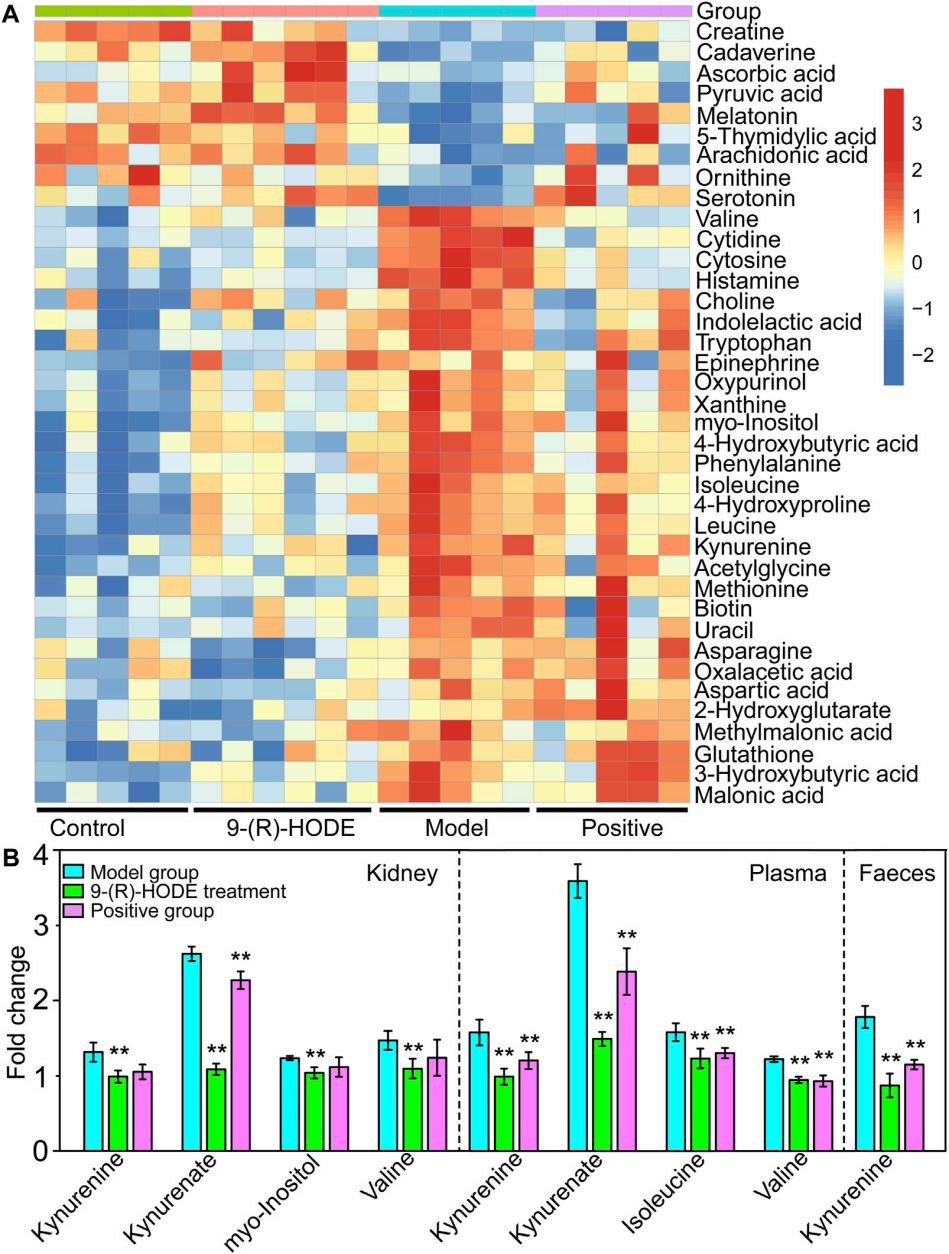
**(A)** The representative extracted heatmap of differential aqueous-soluble metabolites in muscle. The color scale illustrates the relative abundances across the samples. Blue indicates metabolites that were significantly downregulated, while red indicates significantly upregulated metabolites. **(B)** Fold change of various representative metabolites in the model and 9-(R)-HODE treatment groups compared with the control group in kidney, plasma and faeces. *p < 0.05, **p < 0.01, significantly different from the model group.

To explore the changes in metabolites induced by 9-(R)-HODE and disclose its mechanism of hypoglycemic activity and alleviating diabetic complications, LC‒MS/MS-based metabolomic profiling technology was performed for characterization and quantitative detection in tissues, faeces and plasma. The corresponding LC‒MS total ion chromatograms (TICs) in negative and positive ion modes of ESI are shown in Supplementary Figures S1–S3. The PCA and PLS-DA score plots showing global views of the metabolic profiles are shown in Supplementary Figure S4 and Figures 3B,C. The normal, diabetic model, and 9-(R)-HODE treatment groups were basically distinguished from one another in the PCA models of liver, muscle and faeces. A clearer separation among these groups was observed in the PLS-DA models of liver and muscle (Figures 3B,C). Most of the samples of the positive group were closely clustered with the 9-(R)-HODE treatment group, although some of them could not be separated from the diabetic model group. However, no distinct separation among groups could be observed in either PCA or PLS-DA models of aqueous-soluble metabolites in kidney, plasma and faeces. Amino acids, organic acids, alcohols and amines were the most affected by 9-(R)-HODE treatment (Figures 3, 4, 10 and Supplementary Table S3), and the summary of the representative metabolite variations in different conditions was presented in Supplementary Table S3.

#### 3.4.1 Metabolomics study in liver

Distinct patterns of the entire metabolome in the liver affected by 9-(R)-HODE are displayed in heatmaps (Figures 3A, Supplementary Figure S5). The generated heatmap (Figure 3A) was selected to present the effect of 9-(R)-HODE after its treatment by the regulation of a variety of differential metabolites toward the control levels (Supplemetary Table S3). These different metabolites were selected for metabolic pathway analysis with MetaboAnalyst and the Kyoto Encyclopedia of Genes and Genomes online database to uncover the potential mechanisms underlying the antidiabetic efficacy of 9-(R)-HODE at the metabolic level. We identified the main influential metabolic pathways, e.g., ascorbate and aldarate metabolism, lysine degradation, and the citrate cycle (Figure 3D and Supplementary Table S1).

Myo-inositol and its various biochemical derivatives are broadly distributed in mammalian tissues and cells, where they provide important biological functions (Holub, 1986). Myo-inositol is the precursor that can be converted to its isomer chiro-inositol through epimerization (Bizzarri et al., 2016), which can accelerate the dephosphorylation of glycogen synthase and act as a mediator of intracellular insulin action (Larner, 2002). Previous studies have shown that chiro-inositol content decreased in urine and tissues of human subjects and animals with type 2 diabetes, accompanied by an increase in myo-inositol content. The altered inositol excretion patterns were directly related to insulin resistance (Ortmeyer et al., 1993). In our study, myo-inositol displayed an increased content in the diabetic mice but showed a downregulated trend in the group receiving 9-(R)-HODE treatment (Figure 3A, Supplemetary Table S3). The same regulatory trend on myo-inositol was displayed in both kidney and muscle (Figures 4A,B, Supplemetary Table S3).

**FIGURE 5:**
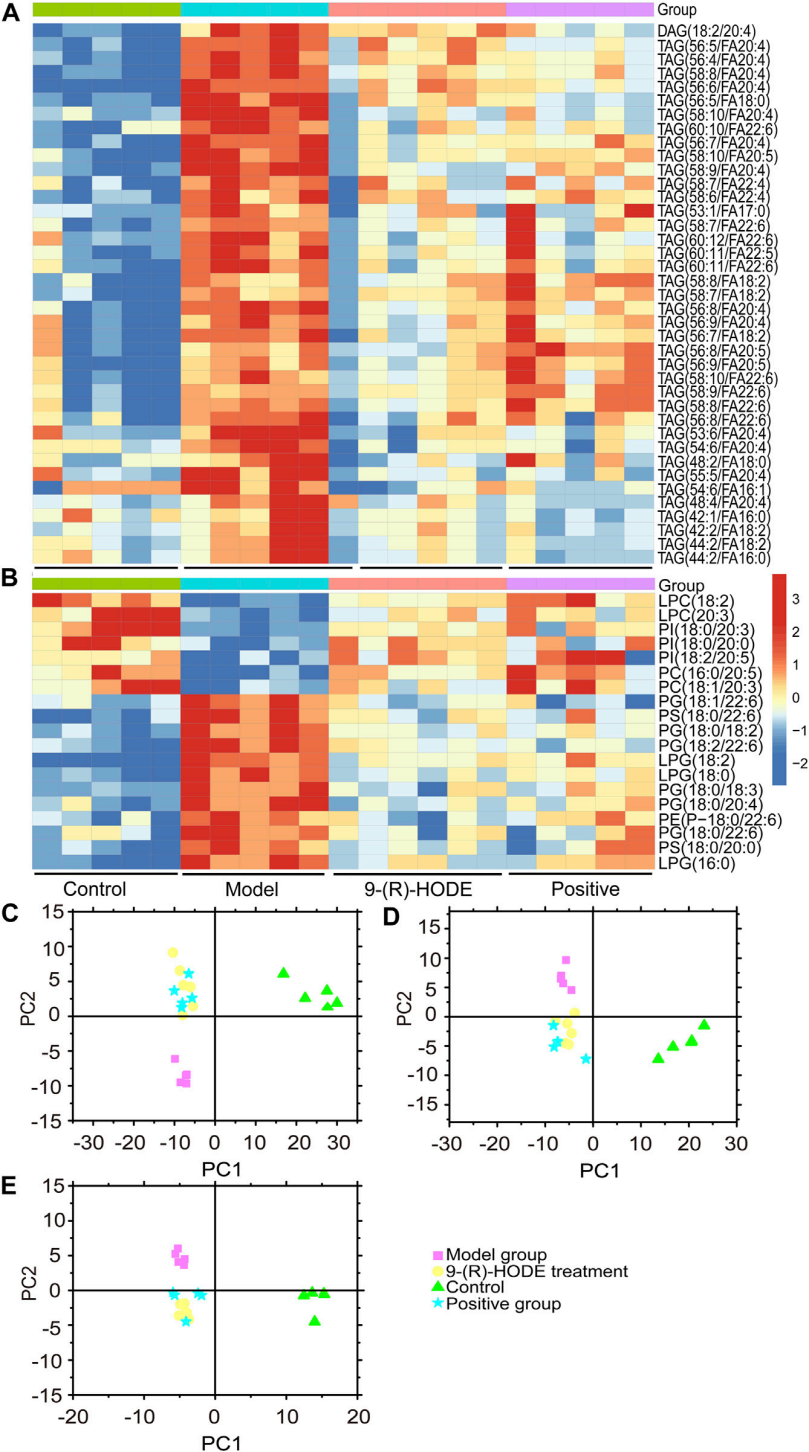
**(A,B)** The representative extracted heatmap of differential lipids in the liver (A: positive mode, B: negative mode). The color scale illustrates the relative abundances across the samples. Blue indicates metabolites that were significantly downregulated, while red indicates significantly upregulated metabolites. **(C–E)** PLS-DA score plots of PC1 vs. PC2 based on the LC‒MS/MS data of lipids in the liver (C: positive mode, D: negative mode) and kidney (E: negative mode).

**FIGURE 6:**
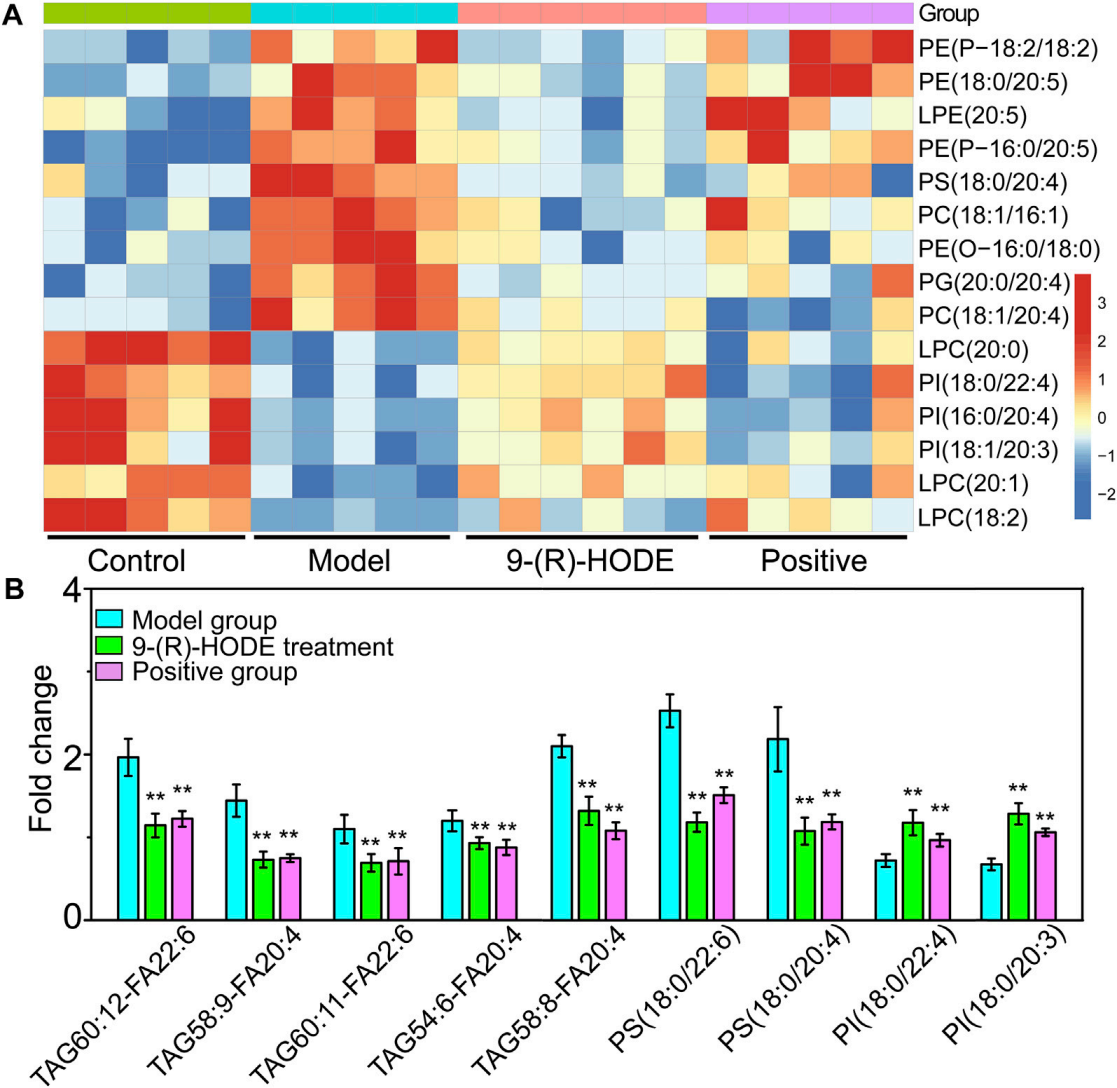
**(A)** The representative extracted heatmap of lipids in the kidney. The color scale illustrates the relative abundances across the samples. Blue indicates metabolites that were significantly downregulated, while red indicates significantly upregulated metabolites. **(B)** Fold change of various representative metabolites in the model and 9-(R)-HODE treatment groups compared with the control group in plasma. *p < 0.05, **p < 0.01, significantly different from the model group.

Inositol exists freely and bound covalently to phospholipids as phosphatidylinositol (PI) in mammalian tissues and cells. PI accounts for 2–12% of the total phospholipids in various mammalian tissues (White, 1973). PI can partially compensate for metabolic abnormalities due to insulin resistance, e.g., by stimulating GLUT4 translocation into the cell membrane, phosphoinositol-3-phosphate increases glucose utilization in tissue cells (Unfer et al., 2017). The concentration of PI decreases in related tissues in diabetic animals (Palmano et al., 1977), and the activities of the enzymes involved in phosphoinositide metabolism decrease (Whiting et al., 1979). In our study, some specific PIs showed an upregulated trend in the group receiving 9-(R)-HODE treatment (Figure 5B, Figure 6A).

Diabetes is not able to use glucose efficiently, and glucose may be shunted in increased quantities into noninsulin-sensitive pathways in the diabetic state. Previous studies have suggested that the relative contribution of the glucuronic acid pathway to total CO_2_ production from glucose is increased in diabetic rabbits (Hinohara et al., 1974). It has been reported that urinary excretion of glucuronic acid (Müting, 1964) is markedly increased in diabetic patients and that glucose catabolism *via* the glucuronic acid pathway is greater in adipose tissue from alloxan diabetic rats than from normal rats (Winegrad and Burden, 1965) due to the enhanced activity of both UDPGA-pyrophosphatase and D-glucuronic acid-1-phosphate phosphatase (Hinohara et al., 1974). Moreover, endogenous glucuronic acid can form in the living body *via* the oxidation of myo-inositol (Charalampous and Lyras, 1957). Glucuronic acid displayed a downregulated trend in the group receiving 9-(R)-HODE treatment (Figure 3A, Supplementary Table S3) in our study.

In diabetes, glucose is not used efficiently, which allows the body to rely on alternative energy sources in tissues and other organs. The citrate cycle is the main source of energy for cells, and an elevated citrate cycle can be found in the diabetic mouse liver due to insulin resistance (Satapati et al., 2012). The downregulation of oxalacetic acid and malic acid involved in the citrate cycle in the 9-(R)-HODE treatment group suggested a recovery of hepatic energy metabolism by using 9-(R)-HODE compared with the model group (Figure 3A, Supplementary Table S3).

Valine, leucine and isoleucine are essential branched chain amino acids (BCAAs) that are responsible for the regulation of growth, protein biosynthesis and metabolism (Millward et al., 2008). Excessive BCAAs are significantly associated with insulin resistance (Newgard, 2012) by the activation of mammalian target of rapamycin complex 1 (mTORC1), which ultimately results in secretory deficiency of β-cells (Lynch and Adams, 2014). Isoleucine and valine in the liver (Figure 3A, Supplementary Table S3) and valine, leucine and isoleucine in the muscle (Figure 4A, Supplementary Table S3) displayed an increased content in the diabetic mice, while a downregulated trend was observed in the group receiving 9-(R)-HODE treatment.

#### 3.4.2 Metabolomics study on muscle

Distinct patterns of the entire metabolome in muscle affected by 9-(R)-HODE are displayed in heatmaps (Figure 4A, Supplementary Figure S6). The metabolic pathway analysis of the representative metabolites revealed that the regulatory effect of 9-(R)-HODE on muscle was primarily focused on alanine, aspartate and glutamate metabolism and tryptophan metabolism (Figure 3E, Supplementary Table S2).

**FIGURE 7:**
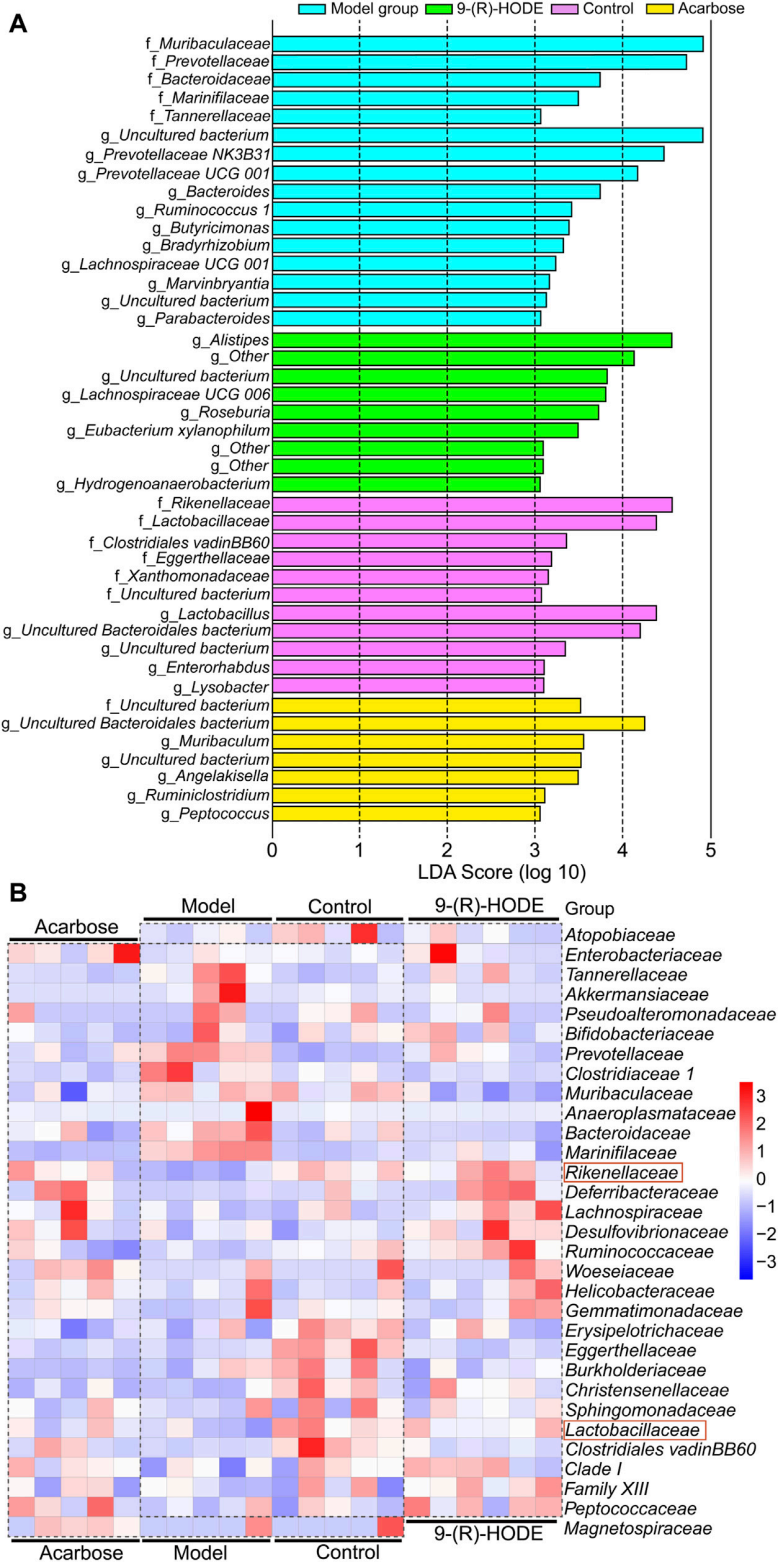
Metabolic network of the significantly altered metabolites involved in 9-(R)-HODE treatment or acarbose. T: 9-(R)-HODE treatment, P: acarbose treatment, C: control group. Green indicates metabolites that were significantly downregulated, red indicates significantly upregulated metabolites, while white indicates no significant changes in metabolites compared with the model mice.

Tryptophan is an essential amino acid, and its metabolism in humans includes protein and nonprotein routes. The major nonprotein route of tryptophan metabolism is the formation of kynurenine, which is catalyzed by rate-limiting enzymes: indoleamine 2, 3-dioxygenase (IDO) or TRP 2, 3-dioxygenase (TDO) (Schwarcz et al., 2012). IDO is activated by inflammatory stimuli, e.g., cytokines and bacterial metabolites (Leclercq et al., 2021). The inhibition of signaling downstream of the insulin receptor is a primary mechanism through which inflammatory signaling leads to insulin resistance (Aguirre et al., 2002). A previous study suggested that inflammation-induced upregulation of tryptophan-kynurenine metabolism, resulting in the excessive production of kynurenine, is one of the factors predisposing patients to insulin resistance (Oxenkrug, 2013). A previous study indicated that kynurenine was negatively associated with gut bacteria of the Ruminococcaceae family (Leclercq et al., 2021) and Lactobacillaceae family (Valladares et al., 2013) and positively associated with Akkermansiaceae family (Leclercq et al., 2021). The anti-inflammatory bacterium *Faecalibacterium prausnitzii* belongs to the Ruminococcaceae family, and *Lactobacillus johnsonii* belongs to the Lactobacillaceae family and has been shown to be negatively correlated with kynurenine and IDO activities (Valladares et al., 2013; Leclercq et al., 2021). In our study, kynurenine content displayed a decreasing trend in muscle, liver, kidney, plasma and faeces in the group treated with 9-(R)-HODE (Figures 3A, 4A,B), while the bacteria of the Ruminococcaceae and Lactobacillaceae families showed an upregulation and the Akkermansiaceae family presented a downregulation compared with the diabetic mice (Figure 7B). Approximately 5% of the nonprotein route of tryptophan metabolism is utilized for the formation of methoxyindoles, e.g., serotonin, *N*-acetylserotonin, and melatonin (Schwarcz et al., 2012; Oxenkrug, 2013). By binding to the related glycogen phosphorylase receptors (5-HT1, 5-HT2A), serotonin leads to the dephosphorylation of glycogen phosphorylase and results in the stimulation of glycogen synthesis (Tudhope et al., 2012). Serotonin and melatonin were upregulated by 9-(R)-HODE treatment in our study (Figures 4A, 3A, Supplementary Table S3). The formation of serotonin was preferred rather than kynurenine by 9-(R)-HODE treatment, where the hypoglycemic effect was probably achieved by indirectly regulating insulin resistance and glycogen synthesis. Oxaloacetate, which is involved in the citrate cycle, was downregulated by 9-(R)-HODE treatment (Figure 4A, Supplementary Table S3), which was in accordance with the results of liver metabolite analysis.

### 3.5 Lipidomic changes induced by 9-(R)-HODE

The lipidomic changes induced by 9-(R)-HODE in tissues and plasma were detected. Representative LC‒MS TICs in negative and positive ion modes of ESI for the different tissues are shown in Supplementary Figures S7, S8. The PCA and PLS-DA score plots showing global views of the metabolic profiles are shown in Figures 5C–E, Supplementary Figure S9. The normal, diabetic model, and 9-(R)-HODE treatment groups were basically distinguished from one another in the PCA models of the liver (both positive and negative mode) and kidney (negative mode). A clearer separation among these groups was observed in the PLS-DA models of the liver and kidney (Figures 5C–E). However, the acarbose and 9-(R)-HODE treatment group clustered together in either PCA or PLS-DA model.

Changes in the levels of multiple lipids, such as phospholipids, lysophospholipids and glycerolipids, were observed (Figures 5, 6, Supplementary Figures S10–S12, Supplementary Table S3). The regulation of PI (18:0/20:3, 18:0/20:0, 18:2/20:5) in the liver and PI (18:0/22:4, 16:0/20:4, 18:1/20:3) in the kidney by 9-(R)-HODE was revealed by heatmaps (Figure 5B, Figure 6A), which have been partly discussed in Section 3.4.1. Previous studies revealed a decrease in lysophosphatidylcholines (LPCs) in diabetic patients (Floegel et al., 2013; Lee et al., 2016). LPC 18:2 and 20:3 in the liver and LPC 18:2, 20:0 and 20:1 in the kidney were upregulated toward the normal control by 9-(R)-HODE (Figure 5B, Figure 6A). Phosphatidylethanolamines (PEs) represent 20–30% of the total phospholipid pool in cellular membranes (Zhan et al., 2021). The PE P-18:0/22:6 in the liver and PE 18:0/20:5, P-18:2/18:2, P-16:0/20:5, and O-16:0/18:0 in the kidney showed a significant increase in the model group, while they displayed a recovery trend toward the control group after 9-(R)-HODE treatment (Figure 5B, Figure 6A). Increased phosphatidylserine (PS) expression contributes to microvascular complications (Bergen et al., 2018) and diabetic kidney disease (Yu et al., 2018) in diabetes. PS 18:0/22:6 and 18:0/20:0 in the liver and PS 18:0/20:4 in the kidney were downregulated compared with the model group by 9-(R)-HODE treatment (Figure 5B, Figure 6A). Phosphatidylglycerols (PGs) are the principal polyglycerophospholipids observed in mammalian tissues and are synthesized *via* the Kennedy pathway (Kiyasu et al., 1963). In our study, the increase in PG mainly occurred in the liver of the diabetic mice, where PG 18:1/22:6, 18:0/18:2, 18:0/18:3, 18:0/22:6, 18:2/22:6, and 18:0/20:4 and LPG 16:0, 18:2, and 18:0 in the liver and PG 20:0/20:4 in the kidney were downregulated compared with the model group treated with 9-(R)-HODE (Figure 5B, Figure 6A). Moreover, TAGs significantly increased in the livers of the diabetic mice and were obviously recovered by 9-(R)-HODE treatment (Figure 5A).

Moreover, kynurenate, myo-inositol and valine in kidney and kynurenate, isoleucine, valine, TAG 60:12-FA 22:6, TAG 58:9-FA 20:4, TAG 60:11-FA 22:6, TAG 54:6-FA 20:4, TAG 58:8-FA 20:4, PS 18:0/22:6, PS 18:0/20:4, PI 18:0/22:4 and PI 18:0/20:3 in plasma displayed the same regulation by 9-(R)-HODE as those in other tissues (Figure 4B, Figure 6B).

### 3.6 SCFA-producing bacteria were increased by 9-(R)-HODE

**FIGURE 8:**
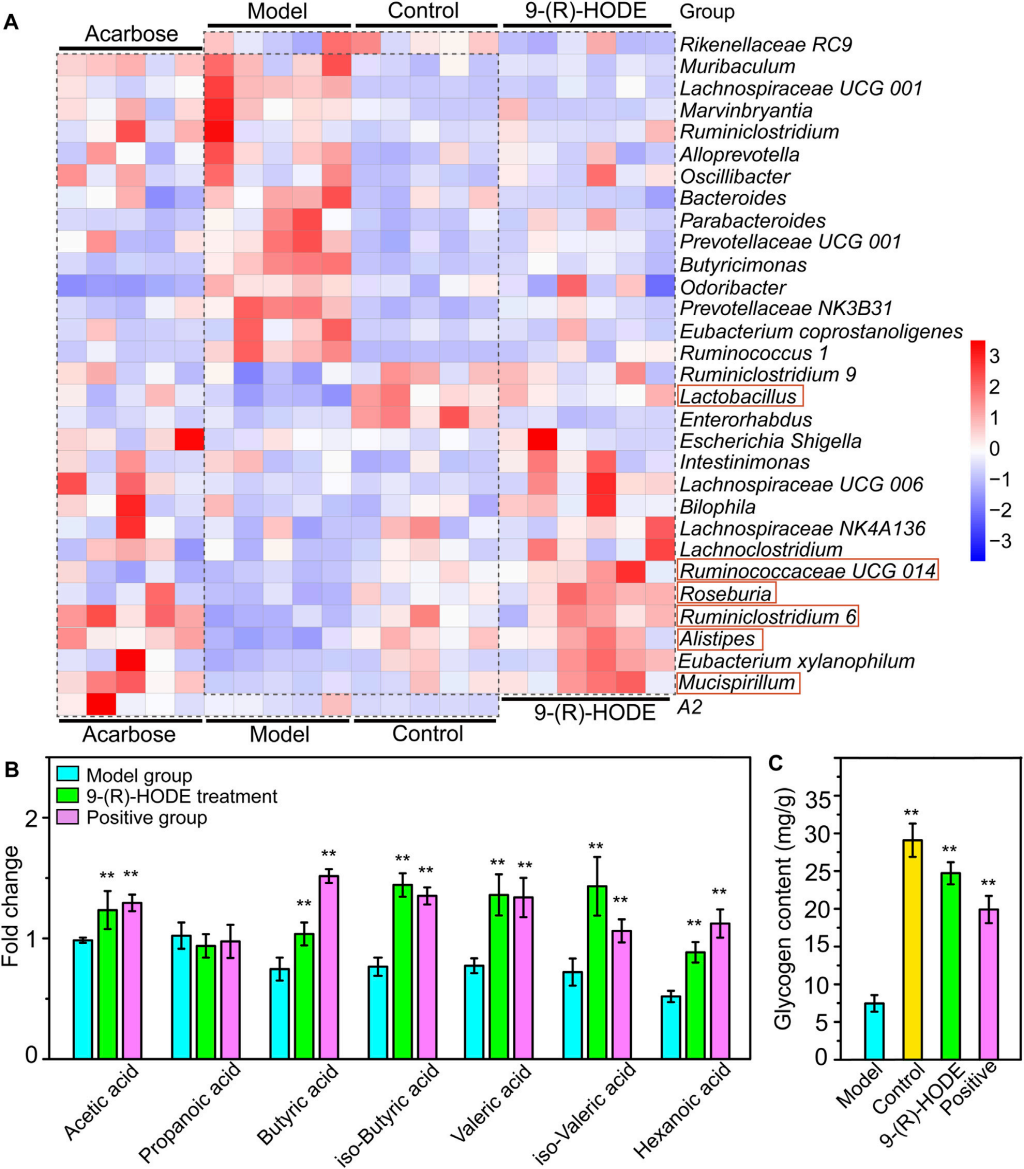
Effect of 9-(R)-HODE and acarbose on gut microbiota. **(A)** Linear discriminant analysis (LDA) scores from LEfSe (LDA effect size) at the family and genus levels. f: At the family level; g: at the genus level. **(B)** Heatmap of the top 30 most abundant differential microbiota at the family level (red rectangles represent SCFA-producing bacteria).

The fecal samples were processed for 16S rDNA sequencing to analyze the differences in gut microbiota. The usable raw reads were obtained with an average of 71,808 reads per sample, and all sequences were divided into 5,715 OTUs based on a 97% similarity level. Based on the sequencing results, the microbial community composition of all samples was annotated. The normal, diabetic model, and 9-(R)-HODE treatment groups showed distinct separation from one another in the PCA model (Supplementary Figure S13). LEfSe was performed to reveal the relative abundances of the differentially abundant bacteria identified. The top 30 dominant bacteria in terms of relative abundance were selected for further analysis. After 9-(R)-HODE treatment, the gut microbiota composition significantly changed at the family and genus levels (Figure 7B, Figure 8A).

At the family level, Rikenellaceae (LDA score = 4.56%) and Lactobacillaceae (LDA score = 4.38%) were the main bacteria in the control group (Figure 7A), while Muribaculaceae (LDA score = 4.91%), Prevotellaceae (LDA score = 4.73%), and Bacteroidaceae (LDA score = 3.73%) were mostly enriched in the model group (Figure 7A). Rikenellaceae was reported to be the SCFA producer (Wang et al., 2020), and its content in the 9-(R)-HODE treatment group was greater than that in the model group (Figure 7B). Lactobacillaceae underwent a decrease in the diabetic mouse group, which is associated with glucagon-like peptide 1 (GLP-1) resistance (Grasset et al., 2017), and the reduction was also related to chronic renal failure (Vaziri et al., 2016). The heatmap showed that Lactobacillaceae had the lowest content in the model group, while a slight upregulation was observed after 9-(R)-HODE treatment (Figure 7B). The upregulation of Bacteroidaceae was observed in diabetic mice and may cause increased diabetic hyperglycemia due to its capacity to digest cellulose and produce glucose (Zhang et al., 2020), which was reversed by 9-(R)-HODE treatment (Figure 7B). At the genus level, LEfSe results indicated *Lactobacillus* (LDA score = 4.38%), *uncultured Bacteroidales bacterium* (LDA score = 4.19%), *Enterorhabdus* (LDA score = 3.10%), and *Lysobacter* (LDA score = 3.09%) were enriched in the control group. *Alistipes* (LDA score = 4.55%), uncultured genus from Lachnospiraceae family (LDA score = 3.82%), Lachnospiraceae *UGG 006* (LDA score = 3.72%) and *Roseburia* (LDA score = 3.72%) were enriched in the 9-(R)-HODE treatment group, while uncultured genus from Muribaculaceae (LDA score = 4.90%), Prevotellaceae *NK3B31* (LDA score = 4.46%), Prevotellaceae *UCG 001* (LDA score = 4.16%), *Bacteroides* (LDA score = 3.73%), *Ruminococcus 1* (LDA score = 3.41%), *Butyricimonas* (LDA score = 3.38%), *Bradyrhizobium* (LDA score = 3.32%), and Lachnospiraceae *UCG 001* (LDA score = 3.23%) were enriched in the model group (Figure 7A). Previous studies indicated a greater abundance of *Muribaculum* (Chai et al., 2021), *Bacteroides* (Chen et al., 2017), Prevotellaceae *NK3B31* (Wei et al., 2018), *Ruminococcus 1* (Wei et al., 2018), the inflammatory bacteria *Butyricimonas* (Kulkarni et al., 2021) and Lachnospiraceae *UCG 001* (Song et al., 2019), and a lower abundance of *Roseburia* (Qin et al., 2012), in diabetic patients and animal models, which is consistent with our results. The abundances of the above strains were reversed by 9-(R)-HODE treatment (heatmap Figure 8A). Moreover, the heatmap revealed that the families Lactobacillaceae (Ikeyama et al., 2021), *Ruminiclostridium 6* (Yang et al., 2021), Ruminococcaceae *UCG 014* (Tian et al., 2019), *Mucispirillum* (Wang et al., 2020), *Lactobacillus* (LeBlanc et al., 2017)*, Alistipes* (Oliphant and Allen-Vercoe, 2019), and *Roseburia* (Qin et al., 2012), which are potential SCFA producers, were upregulated by 9-(R)-HODE treatment (Figure 7B, Figure 8A).

### 3.7 SCFAs were increased by 9-(R)-HODE

The GC‒MS method was used to quantify the quantity of the main SCFAs in the colon, including acetic acid, propanoic acid, butyric acid, isobutyric acid, valeric acid, isovaleric acid and hexanoic acid. Except for propanoic acid, the other six SCFAs were increased by 9-(R)-HODE treatment (Figure 8B). Moreover, metabolomic data regarding the regulation of butyric acid, isobutyric acid, valeric acid and isovaleric acid in plasma (Supplementary Figure S14) were consistent with the above results.

### 3.8 DESI‒MSI analysis of tissue metabolites

**FIGURE 9:**
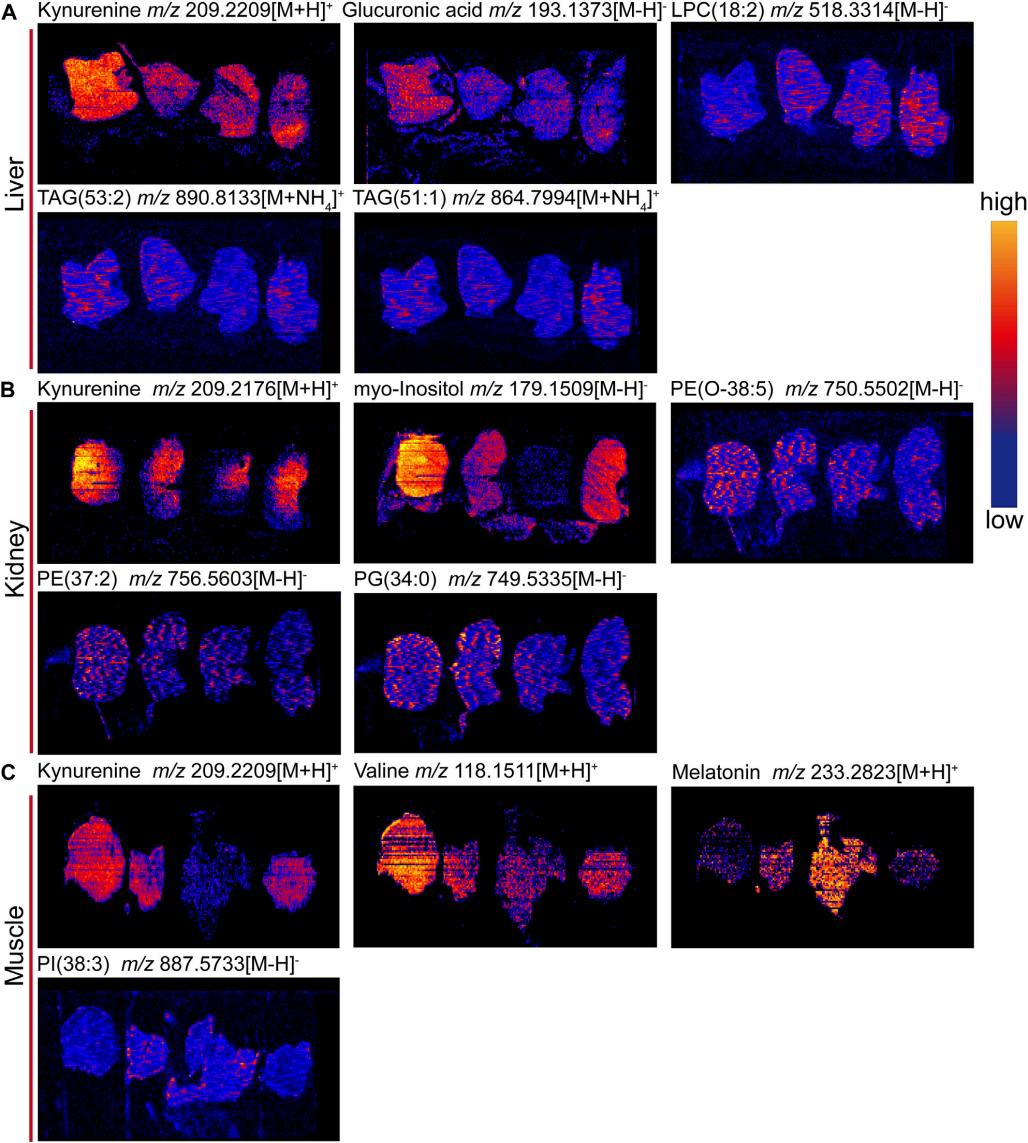
**(A)** Heatmap of the top 30 most abundant differential microbiota at the genus level (red rectangles represent SCFA-producing bacteria). **(B)** SCFA abundances in the mouse colon content. **(C)** Glycogen content in the liver. *p < 0.05, **p < 0.01, significantly different from the model group.

To confirm the above metabolomic analysis, molecular images of frozen sections of the above tissues, i.e., liver, kidney and muscle collected from the model, positive, control and 9-(R)-HODE treatment group mice (Figure 9) were inspected using DESI‒MSI. The TIC spectra of liver, kidney and muscle are shown in Supplementary Figures S15–S17, through which the spatial distribution patterns of more than 1,000 ions were detected. A number of molecules appeared to have different distributions in the tissues among the various groups. In liver sections, kynurenine [*m/z* 209.2209, (M + H)^+^], glucuronic acid [*m/z* 193.1373, (M-H)^-^], and TAG molecules [*m/z* 890.8133, (M + NH_4_)^+^; *m/z* 864.7994, (M + NH_4_)^+^] showed obvious upregulation in the model group, while 9-(R)-HODE treatment reversed this trend compared with that in the control group. LPC [*m/z* 518.3314, (M-H)^-^] was downregulated by 9-(R)-HODE treatment. In the kidney slices, kynurenine [*m/z* 209.2176, (M + H)^+^], myo-inositol [*m/z* 179.1559, (M-H)^-^], PG [*m/z* 749.5335, (M-H)^-^], PE [*m/z* 750.5502, (M-H)^-^], and PE [*m/z* 756.5603, (M-H)^-^] were downregulated by 9-(R)-HODE treatment compared with the model group. In the muscle slices, the abundances of kynurenine [*m/z* 209.2209, (M + H)^+^] and valine [*m/z* 118.1511, (M + H)^+^] in the model group were greater, while those of melatonin [*m/z* 233.2823, (M + H)^+^] and PI [*m/z* 887.5733, (M-H)^-^] were lower, which were reversed by 9-(R)-HODE treatment.

### 3.9 Liver glycogen content determination

To further explore the effect of 9-(R)-HODE on glucose metabolism and to validate the metabolomic mechanism based on metabolite analysis, we examined liver glycogen content. With 9-(R)-HODE treatment, the liver glycogen content was significantly increased (Figure 8C), which confirmed the regulatory effect involved in the pathway analysis based on the different metabolites related to glucose metabolism induced by 9-(R)-HODE.

## 4 Discussion

Our results demonstrate that 9-(R)-HODE may have potent efficacy in ameliorating hyperglycemia and alleviating diabetic complications in diabetic mice. The trajectories of FBG indicated that it was not significantly regulated by 9-(R)-HODE in an initial stage but was downregulated over a longer time span. The lower increased Δblood glucose level of PPG at 1.0 h and 1.5 h indicated the effect of 9-(R)-HODE on regulating postprandial blood glucose, which confirmed the results from our previous *in vitro* study (Liu et al., 2021). The body weight of the diabetic mice displayed a continuous decrease in this study, probably due to the lack of glucose utilization, which is compensated by the increased decomposition of fat and protein. Treatment with 9-(R)-HODE restored the state of emaciation in diabetic mice.

The biochemical parameters indicated that abnormal levels of plasma lipids occurred in diabetic mice, as the TC and LDL-C contents in the model group were significantly higher while the HDL-C contents in the model group were significantly lower than those in the control group, which was consistent with previous observations (Wang et al., 2018). Treatment with 9-(R)-HODE significantly ameliorated the plasma lipid disorder. Diabetes is accompanied by defects in protein and lipid metabolism, thereby leading to many severe complications, including vasculopathy, neuropathy, retinopathy, hepatopathy and nephropathy (Mohamed et al., 2016). Diabetic kidney disease is a common complication of diabetes. Approximately 30% and 40% of patients with type 1 and type 2 diabetes mellitus develop DKD, respectively (Collins et al., 2011). As presented in our data, the decreased CRE, UA and UREA levels and the increased TP levels in the 9-(R)-HODE treatment group indicated the potential ability of 9-(R)-HODE to alleviate kidney dysfunction in diabetic mice, which showed an even better effect than acarbose. Pathological changes were observed in the renal tissue of diabetic mice and were reversed by treatment with 9-(R)-HODE, which was in accordance with previous biochemical parameter descriptions.

**FIGURE 10:**
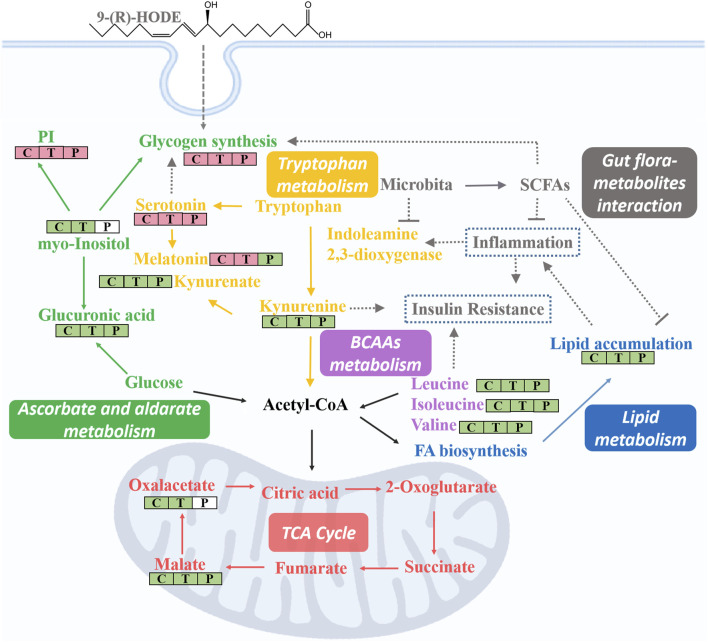
Representative images of tissue metabolite ions detected by desorption electrospray ionization-mass spectrometry imaging (DESI‒MSI). **(A)** Ion images of kynurnine, glucuronic acid, LPC(18:2), TAG (53:2), and TAG (51:1) in livers. **(B)** Ion images of kynurenine, myo-inositol, PE (O-38:5), PE (37:2) and PG (34:0) in kidneys. **(C)** Ion images of kynurnine, valine, melatonin and PI(38:3) in muscles. +: positive mode and -: negative mode. In each image, sections are ordered from left to right by model group, 9-(R)-HODE treatment group, control group and positive group. Scale bar is 4 mm. All ion images are normalized to the TIC, and the blue-to-yellow gradient color scheme refers to low-to-high abundance.

Subsequent metabolomic studies indicated that 9-(R)-HODE induced metabolomic alterations primarily by affecting the levels of amino acids, organic acids, alcohols and amines involved in ascorbate and aldarate metabolism, lysine degradation, and the citrate cycle in the liver but in alanine, aspartate and glutamate metabolism and tryptophan metabolism in muscle. The molecules regulated by 9-(R)-HODE treatment were related to amino acid metabolism, glucose metabolism and energy metabolism (Figure 10) and the metabolites kynurenine (Oxenkrug, 2013), myo-inositol (Ortmeyer et al., 1993) and the branched chain amino acids leucine, isoleucine and valine (Newgard, 2012), which could affect insulin resistance. By mediating the related metabolism or single molecules related to insulin resistance, as mentioned above, 9-(R)-HODE achieved its hypoglycemic effect and alleviated diabetic complications. Moreover, the mediation of kynurenine with 9-(R)-HODE treatment displayed the same trend in kidney, plasma and faeces as in liver and muscle, indicating a systematic effect. The positive group could not be separated from the diabetic model group in the PCA or PLS-DA models of aqueous-soluble metabolites in tissue, plasma and faeces. Although most of the metabolites regulated by 9-(R)-HODE treatment displayed the same regulatory trend by the invention of acarbose, different levels of regulation existed (Figures 3, 4, Figure 10). In the regulation of tryptophan metabolism, acarbose presented weaker effect on modulating metabolite of melatonin in both liver and muscle, and kynurenine in muscle (Figures 3, 4, Figure 10). Compared with acarbose treatment, the treatment of 9-(R)-HODE also caused greater changes of kynurenine and/or its downstream product of kynurenate in kidney, plasma and feaces. As for ascorbate and aldarate metabolism, the metabolite of myo-inositol had a greater downregulation by acarbose treatment in the liver, while myo-inositol level in the muscle and glucuronic acid level in the liver had a greater downregulation by 9-(R)-HODE treatment (Figures 3, 4, Figure 10). The metabolites of oxalacetic acid and malic acid involved in the citrate cycle, and BCAAs of valine, leucine, and isoleucine could not be well modulated by acarbose as well (Figures 3, 4, Figure 10). There is no doubt that 9-(R)-HODE and acarbose had many similarities in their regulatory effects because they are both α-glucosidase inhibitors. The existed regulatory difference might be due to the higher amount of 9-(R)-HODE used in this study, or the possible existence of certain different action mechanism, which needs further study to verify.

Lipidomic studies revealed that 9-(R)-HODE could reverse the lipid metabolism disorder in diabetic mice mainly by regulating phosphatidylinositols, lysophosphatidylcholines, lysophosphatidylcholines, phosphatidylserine, phosphatidylglycerols, lysophosphatidylglycerols and triglycerides in both tissues and plasma. Previous studies indicated the mechanism leading to the decrease in LPCs in diabetic patients may be due to inflammatory processes (Wallace et al., 2014; Klingler et al., 2016). LPC administration lowered blood glucose levels in diabetic mice (Yea et al., 2009). The potential effect of LPCs may be due to the inhibition of inflammation (Wallace et al., 2014) and the activation of PPARδ-dependent gene expression as lipid signaling molecules (Laganà et al., 2016), thus leading to reduced fatty acid-induced inflammation and achieving antidiabetic effects (Klingler et al., 2016). It was reported that specific PEs were increased in gestational diabetes mellitus (Zhan et al., 2021). PE can be transformed into phosphatidylcholine by phosphatidylethanolamine *N*-methyltransferase, and the ratio of PC:PE is inversely related to insulin sensitivity (Lee et al., 2018). The overexpression of specific PS leads to microvascular complications (Bergen et al., 2018) and diabetic kidney disease (Yu et al., 2018) in diabetes. Few studies on the correlation between PG and diabetes have been reported. Hatch, et al. reported that specific PGs in the primary stage of diabetic rat hearts were increased 1.8-fold (Hatch et al., 1995). Glucose metabolism is closely linked to lipid metabolism. Evidence from recent years has indicated that insulin resistance and diabetes are primarily lipid diseases (Parhofer, 2015). The representative interaction is characterized by increased TAGs and HDL-C, known as diabetic dyslipidemia. Elevated triglycerides may cause elevated levels of free fatty acids, which can directly or indirectly modulate the cascade linking insulin receptors by inducing subclinical inflammation, leading to insulin resistance and β-cell dysfunction (Wellen and Hotamisligil, 2005). Lipid, glucose, and energy metabolism and inflammatory processes are correlated to each other, and the regulatory effect on above abnormal lipid levels in diabetes might be the way of 9-(R)-HODE to achieve its anti-diabetic effect. The map linking the integrated metabolic pathways is displayed in Figure 10. The acarbose treatment group had a lot in common with 9-(R)-HODE treatment group in regulating lipid metabolism, which was consistent with the results of inseparable distribution between the groups in both PCA and PLS-DA models. Interestingly, weaker or no regulation regarding most of the modulated lipids with 9-(R)-HODE treatment was observed by the invention of acarbose (Figures 5A,B, Figure 6A), which was consistent with the metabolomic results.

By activation of the AMPK signaling pathway, SCFAs lead to increased expression of peroxisome proliferator-activated receptor α (PPARα), peroxisome proliferator-activated receptor γ (PPARγ) (Jäger et al., 2007), and hormone-sensitive lipase (HSL) and adipose triglyceride lipase (ATGL) as the main enzymes of lipolysis (Tang et al., 2020). Thus, they reduce lipid accumulation, mainly including triglycerides and free fatty acids. SCFAs can stimulate the secretion of glucagon-like peptide-1 (GLP-1) by activating FFAR2, which leads to an increase in insulin secretion and a decrease in pancreatic glucagon secretion (Barrera et al., 2011; Grasset et al., 2017). Moreover, the activation of FFAR2 causes greater expression of leptin, an aliphatic factor secreted by adipocytes, which can directly promote the synthesis of liver glycogen (Fujikawa et al., 2010). By activating FFAR3, SCFAs can stimulate the secretion of peptide tyrosine-tyrosine (PYY), an enteroendocrine hormone, which can lead to reduced food intake (Batterham et al., 2002). Through the pathways mentioned above, SCFAs are closely related to glucose metabolism. SCFAs can inhibit the secretion of inflammatory cytokines, e.g., TNFα, IL-8, and IL-6, by activating FFAR2 and FFAR3 receptors and upregulate the expression of anti-inflammatory factors (Ohira et al., 2013; Halnes et al., 2017). Thus, SCFAs are closely related to lipid, glucose, and energy metabolism and inflammatory processes, and they themselves are affected by SCFA-producing bacteria. In our study, the gut microbiota analysis and SCFA content measurements of the colon contents of mice were processed. The gut microbiota analysis results indicated that the families Rikenellaceae and Lactobacillaceae and the genera *Ruminiclostridium 6*, Ruminococcaceae *UCG 014*, *Mucispirillum*, *Lactobacillus, Alistipes*, and *Roseburia*, which are potential SCFA producers, were upregulated by 9-(R)-HODE. The contents of acetic acid, butyric acid, isobutyric acid, valeric acid, isovaleric acid and hexanoic acid in the colon were upregulated by 9-(R)-HODE treatment. Moreover, the abundance of butyric acid, isobutyric acid, valeric acid and isovaleric acid in plasma showed the same regulatory trend. Previous studies have revealed that the reduction of SCFA production is associated with type 2 diabetes (Qin, et al., 2012), while upregulation of SCFAs can alleviate type 2 diabetic symptoms (Zhao, et al., 2018), which is consistent with our results. Of the top 30 most abundant differential microbiota at the family and genus levels, 29 were the same with either 9-(R)-HODE or acarbose (Figure 7B, Figure 9A) treatment. However, there were differences in the regulation of gut microbiota, e.g., acarbose displayed a weaker effect on modulating *Muribaculum* and *Bacteroides* and a greater effect on regulating Prevotellaceae *NK3B31*, *Ruminococcus 1*, and *Butyricimonas*. Moreover, *Muribaculum* (LDA score = 3.56%), *Angelakisella* (LDA score = 3.49%), *Peptococcus* (LDA score = 3.06%) were the top enriched genera in the acarbose treatment group, which was different from that in 9-(R)-HODE treatment group. Previous studies indicated there was a greater abundance of *Muribaculum* (Chai et al., 2021) and *Angelakisella* (Wang et al., 2018) in diabetic animal model, and *Angelakisella* has a negative correlation with fecal SCFAs (Qiu et al., 2021). *Peptococcus* plays as a pathogenic bacteria existing in diabetic patients (Dualib et al., 2022) and animal models (Shang et al., 2016). For the SCFA-producing bacteria, acarbose showed similar regulation except a weaker effect on regulating Ruminococcaceae *UCG 014* and *Roseburia*. Again, the regulatory role of acarbose and 9-(R)-HODE was confirmed to be the coexistence of difference and identity. The results of the abundances of kynurenine, glucuronic acid, myo-inositol, valine, melatonin, TAGs, LPC, PG, PEs, and PI regulated by 9-(R)-HODE in the DESI‒MSI analysis were in accordance with our tissue metabolomics and lipidomics results, which strongly validated the metabolomics approach in illustrating the hypoglycemic and diabetic complication-alleviating effect of 9-(R)-HODE.

As illustrated by the integrated pathways, several kinds of molecules, e.g., myo-inositol (Larner, 2002), serotonin (Tudhope et al., 2012), and SCFAs (Fujikawa et al., 2010), are closely related to glycogen synthesis due to different mechanisms. The liver is a predominant metabolic organ as well as a key organ for maintaining glucose homeostasis (Zhao et al., 2015); thus, to validate these results, the abundance of liver glycogen was measured. Interestingly, the results indicated that the glycogen content in diabetic mouse livers (7.5 ± 1.1 mg/g) was approximately one-quarter of that in normal mouse livers (29.1 ± 2.1 mg/g), and this phenomenon was significantly reversed by 9-(R)-HODE treatment (23.7 ± 1.5 mg/g) to normal levels. These results again strongly demonstrated the validity of our tissue metabolomics and lipidomics results.

The 9-(R)-HODE was screened out as a α-glucosidase inhibitor in our previous study (Liu et al., 2021). Although α-glucosidase inhibitory drugs have been used as the first-line oral hypoglycemic agents, they are still underutilized therapeutic agents probably due to their mild-to-moderate gastrointestinal side effects, i.e., flatulence, diarrhoea and abdominal ache (Aoki et al., 2010; Joshi et al., 2015). Searching for α-glucosidase inhibitor with multiple mechanisms and low adverse gastrointestinal effects could be a way to resolve the problem. In this study, 9-(R)-HODE displayed differences in the regulation of gut microbiota and related metabolites compared with acarbose, and this might provide the possibility for its further development as a candidate or precursor substance for treatment diabetes. This dynamic crosstalk between the host and its microbiota is important for achieving and maintaining homeostasis. The microbial inhabitants of the gut and microbiota-derived metabolites influence host’s metabolic processes, and host metabolism can in turn affect the gut flora, thus forming a functional interaction between the gut microbiota and host metabolism (Tremaroli and Bäckhed, 2012). *In vivo*, amino acid, glucose, energy and lipid metabolism interacts with each other. Thus, the revealed biological effects of 9-(R)-HODE in this study might be the integrated result of the interaction between the flora and metabolites caused by 9-HODE as well as its action as a α-glucosidase inhibitor. Moreover, 9-HODE has previously been reported to be a PPARγ agonist (Itoh et al., 2008). PPARγ mainly controls the expression of gene networks involved in lipid metabolism (Jäger et al., 2007; Tontonoz and Spiegelman, 2008), inflammation (Glass and Saijo, 2010), and the maintenance of glucose homeostasis (Picard and Auwerx, 2002). These critical roles of PPARγ, particularly in glucose homeostasis, make it an attractive drug target for the treatment of diabetes (Martens et al., 2002; Tontonoz and Spiegelman, 2008). In addition, SCFAs were reported to lead to the increased expression of PPARγ (Jäger et al., 2007). Thus, enhancing PPARγ pathway might be an another underlying mechanism of 9-(R)-HODE to improve diabetes. All together, the revealed biological effect of 9-(R)-HODE in this study might be due to the multiple mechanisms including α-glucosidase inhibition, integrated result of the interaction between the flora and metabolites, and PPARγ pathway activation. However, this study has the following limitations, e.g., 1) the STZ-induced mouse was used as diabetic model, which was more closely mimiced as type 1 diabetes. Type 1 diabetes and type 2 diabetes have different pathogenesis, although there have the same pathological phenomena of blood glucose elevation, and therefore multi-model validation should be applied; 2) Although the mechanism of improving glycogen synthesis has been verified at the molecular level in this study https://fanyi.baidu.com/translate?aldtype=16047&query=Bacteroidales+is+an+order+of+bacteria&keyfrom=baidu&smartresult=dict&lang=auto2zh - ##, other metabolic pathways regulated by 9-(R)-HODE require further validation; 3) Higher amount of 9-(R)-HODE was used in this study compared with that of acarbose, thus further study should be conducted in the future to verify the regulatory difference from acarbose was not caused by dose differences; 4) The effect of 9-(R)-HODE on the normal control mice is unknown. Further studies should be processed to confirm and explore the multiple mechanisms of 9-(R)-HODE as well as investigate its side effects.

## 5 Conclusion

This study demonstrates the potent efficacy of 9-(R)-HODE in ameliorating hyperglycemia and alleviating diabetic complications in diabetic mice. Mechanistically, 9-(R)-HODE can modulate the levels of the related molecules of amino acids, organic acids, alcohols, amines, phospholipids, lysophospholipids and glycerolipids involved in lipid, glucose, energy metabolism and inflammatory processes. Furthermore, the regulation of the gut microbiota composition, mainly SCFA-producing bacteria, was associated with the therapeutic effects of 9-(R)-HODE on diabetic mice. Although 9-(R)-HODE had a weaker effect on regulating FBG and PPG than acarbose at the dosage level used in this study, considering its different structure and potential regulatory difference from acarbose, it still has the potential to be further developed as a promising candidate for the treatment of diabetes.

## Data Availability

The original contributions presented in the study are publicly available. This data can be found here: https://figshare.com/search?q=10.6084%2Fm9.figshare.21321213.
