# Supplementary material for: Action mechanism of hypoglycemic principle 9-(R)-HODE isolated from cortex lycii based on a metabolomics approach
Source: Front Pharmacol. 2022 Oct 21;13:1011608. doi: 10.3389/fphar.2022.1011608 (PMC9633664; doi:10.3389/fphar.2022.1011608)
Supplement: Supplementary file 1 [file DataSheet1.docx]

**Supporting Information**

**Action Mechanism of Hypoglycemic Principle 9-(R)-HODE Isolated from Cortex Lycii Based on a Metabolomics Approach**

Yueqiu Liu^a, b^, Xinyi Hu^a^, Wen Zheng^a^, Lu Zhang^a^, Luolan Gui^a^, Ge Liang^a^, Yong Zhang^a^, Liqiang Hu^a^, Xin Li^a^, Yi Zhong^a^, Tao Su^a^, Xin Liu^a^, Jingqiu Cheng^a^, Meng Gong^a*^

^a^ Laboratory of Clinical Proteomics and Metabolomics, Institutes for Systems Genetics, Frontiers Science Center for Disease-Related Molecular Network, National Clinical Research Center for Geriatrics, West China Hospital, Sichuan University, Chengdu, Sichuan, China

^b^ College of Materials and Chemistry & Chemical Engineering, Chengdu University of Technology, Chengdu, China

*^*^ Corresponding author:*

*Meng Gong*

*E-mail: [gongmeng@](mailto:gongmeng@gazi.edu.tr)scu.edu.cn*

**Chemicals and reagents**

The compound 9-(R)-HODE (purity≧99 %) were purchased from HitGen Inc. (Chengdu, China). LC‒MS-grade formic acid, methyl tert-butyl ether (MTBE), acetonitrile, isopropanol, and methanol were purchased from Fisher Scientific (Hampton, NH, USA). LC‒MS grade acetic acid, ammonium formate, ammonium acetate, dichloromethane (CH_2_Cl_2_), GC‒MS grade propyl chloroformate, *n*-hexane, analytical grade acarbose, hematoxylin and eosin (H&E), formalin, streptozotocin (STZ), sodium citrate, citric acid, Sodium hydroxide (NaOH), carboxymethyl cellulose sodium (CMC-Na) and acetic acid, propionic acid, butyric acid, caproic acid-d3 standards were ordered from Sigma-Aldrich (St. Louis, MO, USA). Bacterial genomic DNA Extraction Kit was purchased from Magen Biotech (Guangzhou, Guangdong, China).

**Plasma sample preparation**

For metabolomics, 50 µL of plasma was mixed with 250 µL of spiked methanol by vortexing for 30 min and centrifuging at 17,000 g for 20 min at 4 °C. The supernatant was concentrated under vacuum to dryness and stored at -80 °C for metabolite analysis. Prior to the metabolite analysis, the extracts were reconstituted in 200 µL of mobile phase mixture (solvent A-solvent B, 20:80, v/v). For lipidomics, 25 µL of plasma was mixed with 228 µL of spiked methanol by vortexing for 1 min. And 750 µL MTBE was added and vortexed at 1500 rpm for another 1 min, followed by placing in the dark for 1 h. After centrifugation (14,000 rpm, 10 min, 4 °C), 700 µL of supernatant was transferred into an EP tube with vacuum drying for 3 h. The above dried lipids were redissolved in 200 µL 50% CH_2_Cl_2_/50% CH_3_OH with 10 mM ammonium acetate followed by vortexing (1500 rpm, 10°C, 30 s) and centrifugation (14,000 rpm, 10 min, 4 °C).

**Tissue sample preparation**

For metabolomics, 10 mg of tissue sample, 200 µL of precooled MeOH/H2O (4:1) solution, and 6 steel balls were mixed and homogenized 4 times at 4 °C (30 s each time). Then, 800 µL of spiked methanol solution was added and swirled for 3 min at 1500 rpm (4 °C). The supernatant was collected after centrifugation. The samples were subsequently placed in the refrigerator for 30 min, sonicated in an ice water bath for 10 min, swirled for 3 min and centrifuged at 13300 rpm at 4 °C for 15 min. An 800 µL volume of the supernatant was transferred into an EP tube. Another 500 µL of MeOH/H2O (4:1) solution was added to the residue, swirled for 3 min and centrifuged at 13300 rpm at 4 °C for 15 min. Four hundred microliters of the supernatant was collected and combined with the former supernatant in an EP tube. Lastly, the extracts were concentrated to dryness under a vacuum and stored at -80 °C for metabolite analysis. The extracts were redissolved in 500 µL of HILIC solution (60% acetonitrile with 5 mM ammonium formate), swirled for 3 min, sonicated in an ice water bath for 10 min, and centrifuged at 13300 rpm at 4 °C for 10 min. Then, 150 µL of the supernatant was obtained for metabolite analysis. A mixture containing 30 µL of each sample was prepared as the quality control (QC). For lipidomics, 10 mg of the tissue from each sample together with 6 steel balls and 200 µL pre-cooled 80% CH_3_OH were mixed three times for tissue homogenization (30 s each time). Then, 150 µL of spiked methanol solution was added and swirled for 3 min at 1500 rpm (4 °C). MTBE (1000 µL) was added and vortexed at 1500 rpm for another 1 min, followed by placing in the dark for 30 min. Thereafter, 250 µL of double deionized water was added and vortexed at 1500 rpm for 1 min, followed by stratification for 10 min in the dark. After centrifugation (14,000 rpm, 10 min, 4 °C), 800 µL of supernatant was transferred into an EP tube with vacuum drying for 3 h. The above dried lipids were redissolved in 200 µL 50% CH_2_Cl_2_/50% CH_3_OH with 10 mM ammonium acetate followed by vortexing (1500 rpm, 10°C, 30 s) and centrifugation (14,000 rpm, 10 min, 4 °C). A mixture containing 30 µL of each sample was prepared as the quality control (QC).

**UPLC‒MS/MS conditions for plasma and faeces metabolite profiling**

Plasma and faeces metabolic profiling analysis were performed on an Ultimate 3000 rapid separation liquid chromatograph coupled with a Q Exactive Plus Q-Orbitrap HRMS (Thermo Fisher Scientific, Waltham, MA, USA) by using a BEH Amide column (2.1 × 100 mm, 1.7 m) (Waters, Milford, MA, USA). For the chromatographic separation, the mobile phase was composed of phase A with 0.2% acetic acid and 10 mM ammonium formate in water and phase B with 90% acetonitrile in water (including 0.15% formic acid and 10 mM ammonium formate). The gradient elution was as follows: 0~2 min, 100% B; 2~9 min, 100~85% B; 9~14 min, 85~50% B; 14~17 min, 50% B for column cleaning; and 17~25 min, 100% B for column re-equilibration. The flow rate was 0.3 mL/min, the column temperature was maintained at 40 °C, and the sample injection volume was 2 µL. The ion source parameters were as follows: sheath gas flow at 35 arb, auxiliary gas flow at 10 arb with a heater temperature at 350 °C, s-lens RF level at 60%, capillary temperature at 320 °C, ion spray voltage at + 3200 V for positive mode and - 3200 V for negative mode. For lipidomic analysis of the plasma samples, the conditions of LC‒MS/MS were the same as those for tissue detection.

**UPLC**‒**MS/MS conditions for tissue metabolite profiling**

Tissue metabolic profiling analysis was performed on a Shimadzu LC-30A liquid chromatograph (Shimadzu, Kyoto, Japan) with coupled with an AB Sciex 5500 triple quadrupole mass spectrometer (AB SCIEX, Framingham, USA). For the aqueous-soluble metabolite analysis, a total of 228 metabolites (99 in positive mode and 128 in negative mode) chosen for the targeted analysis represented the major metabolic pathways in the diabetic alleviation effect when using multiple reaction monitoring mode. For chromatographic separation, a Waters Acquity UPLC BEH Amide column (2.1 mm×100 mm, 1.7 µm, Waters, Milford, USA) was used and the mobile phase was composed of phase A with 10% acetonitrile in water (containing 0.2% acetic acid and 10 mM ammonium acetate) and phase B with 90% acetonitrile in water (including 0.2% acetic acid and 10 mM ammonium acetate). The gradient elution was as follows: 0.0~0.5 min: 0~10% A; 0.5~1.5 min: 10% A; 1.5~5.0 min: 10~55% A; 5.0~10.0 min: 55% A; 10.0~12.0 min: 55~10% A; and 12.0~25.0 min: 10% A. The flow rate was 0.3 mL/min, the column temperature was maintained at 40 °C, and the sample injection volumes were 5 µL and 15 µL for positive and negative modes, respectively. The ion source parameters were as follows: sheath gas flow at 50 psi, aux gas flow at 40 psi, curtain gas flow at 35 psi, interface capillary temperature at 650 °C, ion spray voltage at + 4000 V for positive mode and - 4000 V for negative mode.

For lipidomics, a total of 1251 lipids (635 in positive mode and 616 in negative mode) represented major metabolic pathways were detected. For the positive mode, the Phenomenex Kinetex C18 column (2.1 mm × 100 mm, 2.6 µm, Phenomenex, USA) was operated at 35°C. The injection volume was 2 µL. HPLC conditions include a flow rate of 300 µL/min with a mobile phase of (A) acetonitrile, MeOH and water (1:1:1, v/v/v) containing 7 mM ammonium acetate) and (B) isopropanol including 7 mM ammonium acetate. The gradient elution was as follows: 0.0~1.5 min: 80~60% A; 1.5~3.0 min: 60~40% A; and 3.0~13.0 min: 40~2% A. The MS interface capillary was kept at 550˚C with a sheath gas flow of 55 psi and aux gas flow of 55 psi. The spray voltage was 5.2 kV. For negative mode, the Phenomenex Luna NH_2_ column (2.0 mm × 100 mm, 3 µm, Phenomenex, USA) was operated at 35°C. The injection volume was 2 µL. HPLC conditions include a flow rate of 200 µL/min (0.0~2.0 min) and 200 µL/min (2.01~15.0 min) with a mobile phase of (A) 7% dichloromethane in acetonitrile (containing 5 mM ammonium acetate) and (B) 50 % acetonitrile in water (containing 2 mM ammonium acetate, pH=8.2). The gradient elution was as follows: 0.0~2.0 min: 100% A; 2.0~2.01 min: 100% A; 2.01~11.0 min: 100~50% A; 11.0~11.5 min: 50~30% A; 11.5~12.5 min: 30~0% A; and 12.5~15.0 min: 0% A. The MS interface capillary was kept at 550˚C with a sheath gas flow of 50 psi and aux gas flow of 50 psi. The spray voltage was -4.5 kV.

**LC**‒**MS conditions for SCFAs measurement**

Derivatives were separated using an DB-5MS capillary column (50 m, i.d. 0.25 mm film thickness, Agilent J & W Scientific, Folsom, CA). One microliter of derivatives was injected in split mode with a ratio of 10:1, and the solvent delay time was set to 5 min. Helium was used as a carrier gas at a constant flow rate of 1 mL//min through the column. The initial oven temperature was held at 50°C for 2 min, ramped to 70°C at a rate of 10°C/min , to 85°C at a rate of 3°C/min, to 110°C at a rate of 5°C/min, to 290°C at a rate of 30°C/min, and finally held at 290°C for 8 min. The temperatures of the front inlet, transfer line, and electron impact (EI) ion source were set at 260, 290, and 230°C, respectively. The electron energy was -70 eV, and the mass spectral data was collected in a full scan mode (m/z 30~600).

**Statistical analysis**

The raw metabolic profile data were imported into MultiQuant (v2.0.3) for integration. The metabolites were identified with available reference standards in our lab and the web-based resources, e.g., the Human Metabolome Database (http://www.hmdb.ca/), METLIN (http:// metlin.scripps.edu/index.php) data source, and LIPID MAPS ([https://www.lipidmaps.org/](https://www.lipidmaps.org/)). The pretreated data, including the sample information, metabolite identity, and peak intensities, were then processed in R software (v3.5.1, Vienna, Austria, https://www.r-project.org/), in which compounds with missing values > 50% and coefficients of variation (CVs) > 20% for metabolites in the QC samples were filtered and treated with the k-nearest neighbor algorithm for missing value imputation. Subsequently, a principal component analysis (PCA) and partial least squares discrimination analysis (PLS-DA) were performed after median normalization to discriminate among samples with different treatments. The heatmaps were drawn with R software. A metabolic pathway analysis was performed by using MetaboAnalyst 5.0 (Quebec, Canada, https://www.metaboanalyst.ca/home.xhtml) according to the pathway topology analysis together with pathway enrichment analysis through the Kyoto Encyclopedia of Genes and Genomes (www.genome.jp/kegg/) database to identify the representative metabolic pathways. The Wilcox test was performed to compare the data between two groups and the Kruskal-Wallis test was performed to investigate alterations among multiple groups.

**Desorption electrospray ionization-mass spectrometry imaging (DESI‒MSI)**

Liver, kidney and muscle tissues from all the treatment groups were sliced frozen to obtain sections with a thickness of 8 µm. The mass spectrometry imaging profiles were performed with a DESI (2D, Indianapolis, Prosolia, USA) source coupled to an MS (Synapt G2-Si, Waters, Milford, USA). DESI‒MSI was performed in both negative and positive modes. The relative parameters were set as follows: spatial resolution at 50 µm (spray spot diameter) for liver samples, 80 µm for kidney and muscle samples; scanning range from 50 to 1200 m/z; histologically compatible solvent (95% CH_3_OH/5% H2O with 0.1% formic acid) at a flow rate of 2 µL/min; voltage at 5 kV for positive mode and -4.5 kV for negative mode; and sheath gas flow at a 0.5 Mbar N_2_ flow was applied for electrospray nebulization. The samples were scanned under impinging charged droplets on a 2D moving stage in horizontal rows where the inlet-to-surface distance was set to 2 mm, the spray incident angle was set to 60°, and the spray-to-inlet distance was set to 5 mm. The transformation of the raw data to image files was performed using HDI v1.5 (Waters, Milford, USA).

**Figure Legends**

**Figure S1.** Typical LC‒MS/MS TIC spectra of tissue metabolic profiles. Pos: LC‒MS/MS detection with positive mode, neg: LC‒MS/MS detection with negative mode.

**Figure S2.** Typical LC‒MS/MS TIC spectra of plasma metabolic profiles.

**Figure S3.** Typical LC‒MS/MS TIC spectra of faeces metabolic profiles.

**Figure S4.** (A-E) PCA score plots of PC1 vs. PC2 based on the LC‒MS/MS data of aqueous-soluble metabolites in the liver (A), kidney (B), muscle (C), plasma (D) and faeces (E).

**Figure S5.** The heatmap of differential aqueous-soluble metabolites in the liver. The color scale illustrates the relative abundances across the samples. Blue indicates metabolites that were significantly downregulated, while red indicates significantly upregulated metabolites.

**Figure S6.** The heatmap of differential aqueous-soluble metabolites in the muscle. The color scale illustrates the relative abundances across the samples. Blue indicates metabolites that were significantly downregulated, while red indicates significantly upregulated metabolites.

**Figure S7.** Typical LC‒MS/MS TIC spectra of tissue lipids. Pos: LC‒MS/MS detection with positive mode, neg: LC‒MS/MS detection with negative mode.

**Figure S8.** Typical LC‒MS/MS TIC spectra of plasma lipids.

**Figure S9.** (A-G) PCA score plots of PC1 vs. PC2 based on the LC‒MS/MS data of lipids in the liver (A: negative mode, B: positive mode), kidney (C: negative mode, D: positive mode), muscle (E: negative mode, F: positive mode) and plasma (G).

**Figure S10.** The heatmap of differential lipids in the liver (negative mode). The color scale illustrates the relative abundances across the samples. Blue indicates metabolites that were significantly downregulated, while red indicates significantly upregulated metabolites.

**Figure S11.** The heatmap of differential lipids in the liver (positive mode). The color scale illustrates the relative abundances across the samples. Blue indicates metabolites that were significantly downregulated, while red indicates significantly upregulated metabolites.

**Figure S12.** The heatmap of differential lipids in the kidney (negative mode). The color scale illustrates the relative abundances across the samples. Blue indicates metabolites that were significantly downregulated, while red indicates significantly upregulated metabolites.

**Figure S13.** PCA score plots of PC1 vs. PC2 based on the gut microbiota data of colon content.

**Figure S14.** SCFA abundances in the mouse plasma. **P* < 0.05, ***P* < 0.01, significantly different from the model group.

**Figure S15.** Typical DESI‒MSI TIC spectra of liver sections including model, 9-(R)-HODE treatment, control and positive group. Pos: detection with positive mode, neg: detection with negative mode.

**Figure S16.** Typical DESI‒MSI TIC spectra of kidney sections including model, 9-(R)-HODE treatment, control and positive group. Pos: detection with positive mode, neg: detection with negative mode.

**Figure S17.** Typical DESI‒MSI TIC spectra of muscle sections including model, 9-(R)-HODE treatment, control and positive group. Pos: detection with positive mode, neg: detection with negative mode.

**Tables**

**Table S1** Metabolite pathway changes in the liver treated with 9-(R)-HODE

**Table S2** Metabolite pathway changes in the muscle treated with 9-(R)-HODE

**Table S3** Summary of the representative metabolite variations in different conditions





Figure S1. Typical LC‒MS/MS TIC spectra of tissue metabolic profiles. Pos: LC‒MS/MS detection with positive mode, neg: LC‒MS/MS detection with negative mode.



Figure S2. Typical LC‒MS/MS TIC spectra of plasma metabolic profiles.





Figure S3. Typical LC‒MS/MS TIC spectra of faeces metabolic profiles.

Figure S4. (A-E) PCA score plots of PC1 vs. PC2 based on the LC‒MS/MS data of aqueous-soluble metabolites in the liver (A), kidney (B), muscle (C), plasma (D) and faeces (E).

Figure S5. The heatmap of differential aqueous-soluble metabolites in the liver. The color scale illustrates the relative abundances across the samples. Blue indicates metabolites that were significantly downregulated, while red indicates significantly upregulated metabolites.

Figure S6. The heatmap of differential aqueous-soluble metabolites in the muscle. The color scale illustrates the relative abundances across the samples. Blue indicates metabolites that were significantly downregulated, while red indicates significantly upregulated metabolites.





Figure S7. Typical LC‒MS/MS TIC spectra of tissue lipids. Pos: LC‒MS/MS detection with positive mode, neg: LC‒MS/MS detection with negative mode.





Fig. S8. Typical LC‒MS/MS TIC spectra of plasma lipids.

Figure S9. (A-G) PCA score plots of PC1 vs. PC2 based on the LC‒MS/MS data of lipids in the liver (A: negative mode, B: positive mode), kidney (C: negative mode, D: positive mode), muscle (E: negative mode, F: positive mode) and plasma (G).

Figure S10. The heatmap of differential lipids in the liver (negative mode). The color scale illustrates the relative abundances across the samples. Blue indicates metabolites that were significantly downregulated, while red indicates significantly upregulated metabolites.

Figure S11. The heatmap of differential lipids in the liver (positive mode). The color scale illustrates the relative abundances across the samples. Blue indicates metabolites that were significantly downregulated, while red indicates significantly upregulated metabolites.

Figure S12. The heatmap of differential lipids in the kidney (negative mode). The color scale illustrates the relative abundances across the samples. Blue indicates metabolites that were significantly downregulated, while red indicates significantly upregulated metabolites.

Figure S13. PCA score plots of PC1 vs. PC2 based on the gut microbiota data of colon contents.

Figure S14. SCFA abundances in the mouse plasma. **P* < 0.05, ***P* < 0.01, significantly different from the model group.





Figure S15. Typical DESI‒MSI TIC spectra of liver sections including model, 9-(R)-HODE treatment, control and positive group. Pos: detection with positive mode, neg: detection with negative mode.





Figure S16. Typical DESI‒MSI TIC spectra of kidney sections including model, 9-(R)-HODE treatment, control and positive group. Pos: detection with positive mode, neg: detection with negative mode.





Figure S17. Typical DESI‒MSI TIC spectra of muscle sections including model, 9-(R)-HODE treatment, control and positive group. Pos: detection with positive mode, neg: detection with negative mode.

**Table S1 Metabolite pathway changes in the liver treated with 9-(R)-HODE**

| No. | Pathway name | Match Status^a^ | p | -log(p) | Impact | Hits^b^ |
| --- | --- | --- | --- | --- | --- | --- |
| a | [Ascorbate and aldarate metabolism](https://www.metaboanalyst.ca/MetaboAnalyst/Secure/pathway/ResultView.xhtml) | [2/8](https://www.metaboanalyst.ca/MetaboAnalyst/Secure/pathway/ResultView.xhtml) | 0.012 | 1.92 | 0.5 | myo-Inositol, Glucuronic acid |
| b | [Citrate cycle](https://www.metaboanalyst.ca/MetaboAnalyst/Secure/pathway/ResultView.xhtml) | 2/20 | 0.069 | 1.16 | 0.16 | Oxaloacetate, Malate |
| c | [Lysine degradation](https://www.metaboanalyst.ca/MetaboAnalyst/Secure/pathway/ResultView.xhtml) | 2/25 | 0.102 | 0.99 | 0.15 | 2-Aminoadipate Pipecolate; |
| d | [Tryptophan metabolism](https://www.metaboanalyst.ca/MetaboAnalyst/Secure/pathway/ResultView.xhtml) | 2/41 | 0.226 | 0.65 | 0.13 | Kynurenine, Tryptamine |

a, the matched number of metabolites in one pathway; b, the matched metabolites in one pathway.

**Table S2 Metabolite pathway changes in the muscle treated with 9-(R)-HODE**

| No. | Pathway name | Match Status^a^ | p | -log(p) | Impact | Hits^b^ |
| --- | --- | --- | --- | --- | --- | --- |
| e | alanine, aspartate and glutamate metabolism | 4/28 | 0.003 | 2.47 | 0.31 | Aspartate, Asparagine, Oxaloacetate, Pyruvate |
| d | [Tryptophan metabolism](https://www.metaboanalyst.ca/MetaboAnalyst/Secure/pathway/ResultView.xhtml) | 4/41 | 0.013 | 1.87 | 0.37 | Tryptophan, Melatonin, Serotonin, Kynurenine |

a, the matched number of metabolites in one pathway; b, the matched metabolites in one pathway.

**Table S3 Summary of the representative metabolite variations in different conditions**

| Compound | RT | Molecular weight | Liver | | Kidney | | Muscle | | Plasma | |
| --- | --- | --- | --- | --- | --- | --- | --- | --- | --- | --- |
|  |  |  | T-Con | M-Con | T-Con | M-Con | T-Con | M-Con | T-Con | M-Con |
| **Amino acids** | | | | | | | | | | |
| Leucine | 2.272 | 131.101 |  |  |  |  | 0.34 | 1.35 |  |  |
| Isoleucine | 2.497 | 131.1 | -0.14 | 1.03 |  |  | 0.38 | 1.37 | 0.19 | 0.47 |
| Valine | 3.098 | 117.1 | 0.01 | 1.98 | 0.09 | 0.48 | 0.24 | 2.21 | -0.06 | 0.24 |
| Tryptophan | 2.168 | 204.2 |  |  |  |  | 0.56 | 1.89 |  |  |
| **Organic acids** | | | | | | | | | | |
| Oxalacetate | 4.395 | 132.0 | 0.18 | 1.90 |  |  | 0.08 | 1.58 |  |  |
| Malate | 4.750 | 134.0 | 0.15 | 1.14 |  |  |  |  |  |  |
| Glucuronic acid | 4.728 | 194.1 | 0.02 | 1.96 |  |  |  |  |  |  |
| Kynurenic acid | 1.620 | 189.0 | 0.06 | 1.31 | 0.07 | 1.08 |  |  | 0.27 | 1.41 |
| Butyric acid | 1.415 | 88.0451 |  |  |  |  |  |  | -0.18 | -0.60 |
| iso-Butyric acid | 2.705 | 88.0506 |  |  |  |  |  |  | 0.11 | -0.30 |
| Valeric acid | 1.760 | 102.0662 |  |  |  |  |  |  | 0.01 | -0.39 |
| iso-Valeric acid | 4.267 | 102.0751 |  |  |  |  |  |  | 0.29 | -0.24 |
| **Alcohols** | | | | | | | | | | |
| myo-Inositol | 4.468 | 180.0 | 0.28 | 0.54 | 0.02 | 0.56 | 0.63 | 1.87 |  |  |
| **Amines** | | | | | | | | | | |
| Serotonin | 4.457 | 176.1 |  |  |  |  | 0.63 | -1.13 |  |  |
| Melatonin | 3.152 | 232.1 | 1.37 | -0.39 |  |  | 0.26 | -1.34 |  |  |
| Kynurenine | 2.242 | 208.1 | 0.2 | 1.91 | 0.21 | 0.58 | 0.43 | 1.53 | -0.02 | 1.56 |
| **Phosphatidylinositols** | | | | | | | | | | |
| PI (18:0/22:4) | 9.1683 | 914.581 |  |  | -0.86 | -1.80 |  |  | 0.16 | -0.24 |
| PI (16:0/20:4) | 9.2395 | 858.518 |  |  | -1.01 | -1.97 |  |  |  |  |
| PI (18:1/20:3) | 9.2627 | 886.550 |  |  | -1.00 | -2.02 |  |  |  |  |
| PI (18:0/20:3) | 9.1767 | 888.565 | -1.24 | -2.26 |  |  |  |  | 0.23 | -0.26 |
| PI (18:0/20:0) | 9.2424 | 894.612 | -0.99 | -2.43 |  |  |  |  |  |  |
| PI (18:2/20:5) | 7.4108 | 880.503 | 0.21 | -1.21 |  |  |  |  |  |  |
| **Lysophosphatidylcholines** | | | | | | | | | | |
| LPC (18:2) | 6.1560 | 519.346 | -0.43 | -1.81 | -1.90 | -2.08 |  |  |  |  |
| LPC (20:0) | 6.1825 | 551.409 |  |  | -1.47 | -2.48 |  |  |  |  |
| LPC (20:1) | 6.2011 | 549.393 |  |  | -0.36 | -1.97 |  |  |  |  |
| LPC (20:3) | 6.1126 | 545.362 | -1.06 | -2.04 |  |  |  |  |  |  |
| **Phosphatidylethanolamines** | | | | | | | | | |  |
| PE (18:0/20:5) | 5.9964 | 765.524 |  |  | 0.65 | 1.13 |  |  |  |  |
| PE (P-18:2/18:2) | 6.0326 | 723.5 |  |  | 0.50 | 1.29 |  |  |  |  |
| PE (P-16:0/20:5) | 6.0044 | 721.5 |  |  | 1.22 | 2.17 |  |  |  |  |
| PE (O-16:0/18:0) | 6.6907 | 705.56 |  |  | 0.59 | 1.83 |  |  |  |  |
| PE (P-18:0/22:6) | 5.9358 | 775.5 | 0.51 | 1.73 |  |  |  |  |  |  |
| **Phosphatidylserine** | | | | | | | | | | |
| PS (18:0/20:4) | 5.9146 | 811.529 |  |  | 0.52 | 1.83 |  |  | 0.04 | 0.58 |
| PS (18:0/22:6) | 7.6900 | 835.529 | 0.62 | 2.08 |  |  |  |  | 0.08 | 0.67 |
| PS (18:0/20:0) | 5.5251 | 819.592 | 0.86 | 2.14 |  |  |  |  |  |  |
| **Phosphatidylglycerols** | | | | | | | | | | |
| PG (18:1/22:6) | 6.4441 | 820.518 | 0.16 | 1.29 |  |  |  |  |  |  |
| PG (18:0/18:2) | 6.5993 | 774.534 | 0.70 | 2.38 |  |  |  |  |  |  |
| PG (18:0/18:3) | 6.5959 | 772.518 | 0.60 | 2.14 |  |  |  |  |  |  |
| PG (18:0/22:6) | 6.4595 | 822.534 | 0.43 | 1.84 |  |  |  |  |  |  |
| PG (18:2/22:6) | 6.4376 | 818.503 | 1.08 | 2.38 |  |  |  |  |  |  |
| PG (18:0/20:4) | 6.5653 | 798.534 | 0.56 | 2.21 |  |  |  |  |  |  |
| PG (20:0/20:4) | 5.2410 | 826.569 |  |  | 0.49 | 1.93 |  |  |  |  |
| **Lysophosphatidylglycerols** | | | | | | | | | | |
| LPG (16:0) | 7.5601 | 484.273 | 0.21 | 1.79 |  |  |  |  |  |  |
| LPG (18:2) | 7.4597 | 508.273 | 1.60 | 2.58 |  |  |  |  |  |  |
| LPG (18:0) | 7.4499 | 512.304 | 1.17 | 2.46 |  |  |  |  |  |  |
| **Triglycerides** | | | | | | | | | | |
| TAG56:5-FA20:4 | 10.3235 | 908.8 | 1.52 | 2.51 |  |  |  |  |  |  |
| TAG56:4-FA20:4 | 10.3239 | 910.8 | 1.48 | 2.45 |  |  |  |  |  |  |
| TAG58:8-FA20:4 | 9.9403 | 930.8 | 1.33 | 2.65 |  |  |  |  | 0.22 | 0.79 |
| TAG56:6-FA20:4 | 10.0041 | 906.8 | 1.73 | 2.58 |  |  |  |  |  |  |
| TAG56:5-FA18:0 | 10.2591 | 908.8 | 1.13 | 2.52 |  |  |  |  |  |  |
| TAG58:10-FA20:4 | 9.3798 | 926.8 | 0.33 | 2.35 |  |  |  |  |  |  |
| TAG60:10-FA22:6 | 9.8232 | 954.8 | 0.72 | 2.27 |  |  |  |  |  |  |
| TAG56:7-FA20:4 | 9.6425 | 904.8 | 0.85 | 2.46 |  |  |  |  |  |  |
| TAG58:10-FA20:5 | 9.2293 | 926.8 | 0.94 | 2.48 |  |  |  |  |  |  |
| TAG58:9-FA20:4 | 9.5502 | 928.8 | 0.66 | 2.55 |  |  |  |  | -0.25 | 0.42 |
| TAG58:7-FA22:4 | 9.9565 | 932.8 | 0.74 | 2.06 |  |  |  |  |  |  |
| TAG58:6-FA22:4 | 10.2840 | 934.8 | 0.89 | 2.14 |  |  |  |  |  |  |
| TAG53:1-FA17:0 | 10.7943 | 874.8 | 0.50 | 1.67 |  |  |  |  |  |  |
| TAG58:7-FA22:6 | 10.1923 | 932.8 | 0.72 | 1.83 |  |  |  |  |  |  |
| TAG60:12-FA22:6 | 9.2514 | 950.8 | 0.11 | 1.66 |  |  |  |  | 0.11 | 0.78 |
| TAG60:11-FA22:5 | 9.4037 | 952.8 | 0.67 | 2.14 |  |  |  |  |  |  |
| TAG60:11-FA22:6 | 9.4193 | 952.8 | 0.54 | 2.19 |  |  |  |  | -0.40 | 0.13 |
| TAG58:8-FA18:2 | 9.6547 | 930.8 | 1.08 | 1.71 |  |  |  |  |  |  |
| TAG58:7-FA18:2 | 9.9825 | 932.8 | 1.34 | 1.96 |  |  |  |  |  |  |
| TAG56:8-FA20:4 | 9.2892 | 902.8 | 0.93 | 2.42 |  |  |  |  |  |  |
| TAG56:9-FA20:4 | 8.9351 | 900.8 | 0.84 | 2.04 |  |  |  |  |  |  |
| TAG56:7-FA18:2 | 9.5726 | 904.8 | 0.85 | 2.03 |  |  |  |  |  |  |
| TAG56:8-FA20:5 | 9.3125 | 902.8 | 0.96 | 1.67 |  |  |  |  |  |  |
| TAG56:9-FA20:5 | 8.9309 | 900.8 | 1.07 | 1.80 |  |  |  |  |  |  |
| TAG58:10-FA22:6 | 9.1466 | 926.8 | 1.05 | 1.81 |  |  |  |  |  |  |
| TAG58:9-FA22:6 | 9.5126 | 928.8 | 1.30 | 1.87 |  |  |  |  |  |  |
| TAG56:8-FA22:6 | 9.4514 | 902.8 | 0.43 | 1.77 |  |  |  |  |  |  |
| TAG53:6-FA20:4 | 9.3369 | 864.8 | 0.16 | 1.88 |  |  |  |  |  |  |
| TAG54:6-FA20:4 | 9.5834 | 878.8 | -0.37 | 1.56 |  |  |  |  | -0.06 | 0.19 |
| TAG48:2-FA18:0 | 9.4751 | 802.7 | -0.04 | 1.59 |  |  |  |  |  |  |
| TAG55:5-FA20:4 | 10.1233 | 894.8 | -0.22 | 1.68 |  |  |  |  |  |  |
| TAG54:6-FA16:1 | 9.8419 | 878.8 | -0.86 | 0.93 |  |  |  |  |  |  |
| TAG48:4-FA20:4 | 8.9286 | 798.7 | 0.57 | 1.87 |  |  |  |  |  |  |
| TAG42:1-FA16:0 | 8.3756 | 720.661 | -0.32 | 1.05 |  |  |  |  |  |  |
| TAG42:2-FA18:2 | 8.3820 | 718.645 | 0.27 | 1.66 |  |  |  |  |  |  |
| TAG44:2-FA18:2 | 8.4798 | 746.676 | 0.05 | 1.58 |  |  |  |  |  |  |
| TAG44:2-FA16:0 | 8.4800 | 746.676 | -0.21 | 1.45 |  |  |  |  |  |  |

T: 9-(R)-HODE treatment group; Con: control group; M: model group; RT: retention time.
